# Prefrontal inhibition of threat processing reduces working memory interference

**DOI:** 10.3389/fnhum.2013.00228

**Published:** 2013-05-30

**Authors:** Robert Clarke, Tom Johnstone

**Affiliations:** Centre for Integrative Neuroscience and Neurodynamics, The University of ReadingReading, UK

**Keywords:** emotion, emotion regulation, top-down control, prefrontal cortex (PFC), amygdala, anterior cingulate cortex, anxiety, DCM

## Abstract

Bottom-up processes can interrupt ongoing cognitive processing in order to adaptively respond to emotional stimuli of high potential significance, such as those that threaten wellbeing. However it is vital that this interference can be modulated in certain contexts to focus on current tasks. Deficits in the ability to maintain the appropriate balance between cognitive and emotional demands can severely impact on day-to-day activities. This fMRI study examined this interaction between threat processing and cognition; 18 adult participants performed a visuospatial working memory (WM) task with two load conditions, in the presence and absence of anxiety induction by threat of electric shock. Threat of shock interfered with performance in the low cognitive load condition; however interference was eradicated under high load, consistent with engagement of emotion regulation mechanisms. Under low load the amygdala showed significant activation to threat of shock that was modulated by high cognitive load. A directed top-down control contrast identified two regions associated with top-down control; ventrolateral PFC and dorsal ACC. Dynamic causal modeling provided further evidence that under high cognitive load, top-down inhibition is exerted on the amygdala and its outputs to prefrontal regions. Additionally, we hypothesized that individual differences in a separate, non-emotional top-down control task would predict the recruitment of dorsal ACC and ventrolateral PFC during top-down control of threat. Consistent with this, performance on a separate dichotic listening task predicted dorsal ACC and ventrolateral PFC activation during high WM load under threat of shock, though activation in these regions did not directly correlate with WM performance. Together, the findings suggest that under high cognitive load and threat, top-down control is exerted by dACC and vlPFC to inhibit threat processing, thus enabling WM performance without threat-related interference.

## Prefrontal inhibition of threat processing reduces working memory interference

In daily life we regulate our emotions continuously and automatically in order to remain focused on current thoughts and actions. There must be a balance between the ability to detect and attend to potentially significant, sometimes threatening emotional stimuli, and the ability to focus on current goals without unnecessary interruptions. In typical situations this balance is likely to be maintained automatically with little need for individuals to employ deliberate emotion regulation strategies (Mauss et al., 2007). However, anxiety is associated with reduced top-down control over threat related distractors (Bishop et al., 2004) and deficits in maintaining this balance are apparent in cases of highly anxious individuals where intrusive threat-related perceptions and thoughts severely impact day-to-day activities (Etkin et al., 2010). Interference by emotions and emotional stimuli may be overcome by top-down control mechanisms that either facilitate and protect task-related processing, inhibit the interfering emotional effects or a combination of the two. It is not clear whether overcoming emotional interference occurs with a concomitant regulation of subjective emotion, with the majority of studies using emotional stimuli as opposed to induced emotions *per se*. Here, we examined the interplay between bottom-up threat detection systems and top-down control mechanisms using a spatial WM task performed under threat of electric shock. We also investigated whether individual differences in a completely independent non-emotional attentional control task predict the recruitment of top-down control mechanisms in an emotional control task.

The neural basis of emotion regulation has been primarily studied by explicitly instructing participants to reappraise emotional stimuli (Ochsner et al., 2002; Schaefer et al., 2002; Ochsner and Gross, 2008), implicating a brain network including lateral and ventral prefrontal and cingulate regions. Fewer studies have used tasks in which regulating emotion is required but not explicitly instructed. An example is cognitive tasks performed in the context of emotional distractors or some form of emotion induction, for example anxiety (Dolcos and McCarthy, 2006; Shackman et al., 2006), which can impair task performance (Dolcos and McCarthy, 2006; Shackman et al., 2006; Blair et al., 2007; Oei et al., 2012), particularly for anxious individuals (Fales et al., 2008; Cisler and Koster, 2010). There is some evidence that this interference is reduced when the cognitive load of the task increases (Erthal et al., 2005; Van Dillen and Koole, 2009; Vytal et al., 2012), possibly through the automatic engagement of lateral prefrontal top-down control mechanisms that inhibit subcortical regions involved in emotional responding such as the amygdala (Blair et al., 2007; Van Dillen et al., 2009). However, results are not consistent with other studies reporting greater interference with increasing load (Eysenck et al., 2007).

Lavie's load model (Lavie et al., 2004) attempts to reconcile similar incongruences that exist in the non-emotional cognition and attention domain. Under this framework a distinction between perceptual and cognitive load determines whether distracting stimuli produce interference; under increasing perceptual load fewer resources are available to process the distracting stimuli and so interference is reduced whilst with increasing cognitive load there are fewer cognitive resources available to exert top-down control and so interference increases. It is not clear how such a system may operate for emotional distractors. A straightforward translation of Lavie's model would posit that tasks involving high perceptual load might deplete perceptual resources to such an extent that potentially significant emotional stimuli escape processing, and therefore such tasks do not show effects of interference from emotional stimuli, whereas tasks with high cognitive load will. Indeed, the perceptual load model can account for some results, for example the diminished emotional interference in Erthal et al. (2005) where increased load is perceptual (comparing the orientation of peripherally presented bars) as are the emotional stimuli (negative images). A direct translation of Lavie's model cannot account, however, for a number of studies where emotional interference is diminished by high cognitive load (e.g., Van Dillen and Koole, 2009; Vytal et al., 2012). In addition, emotional stimuli gain preferential processing compared to non-emotional stimuli (Dolan and Vuilleumier, 2003; Alpers and Gerdes, 2007; Stout et al., 2013), and automatic processing of threatening stimuli can lead to increased emotional responding outside of awareness (Whalen et al., 1998; Vuilleumier et al., 2002; Dolan and Vuilleumier, 2003). Therefore, it is unclear whether high perceptual load could reduce perceptual resources to such an extent that emotional stimuli, particularly threatening stimuli, are no longer processed. On the other hand, a model that includes active top-down control allows for the processing and subsequent control of emotional, potentially threatening stimuli.

Many studies suggest top-down control of emotion shares common mechanisms with top-down mechanisms for (non-emotional) attentional control and response inhibition (e.g., Pessoa et al., 2003; Ridderinkhof et al., 2004b; Stevens et al., 2007; Etkin et al., 2011; Shackman et al., 2011). Two specific brain regions, the ventrolateral prefrontal cortex (vlPFC) and dorsal anterior cingulate cortex (dACC) implicated in emotion regulation (Blair et al., 2007; Ochsner and Gross, 2008; Van Dillen et al., 2009) overlap with regions commonly identified in studies of cognitive control (e.g., Herath et al., 2001; Dux et al., 2006). Dorsal ACC is implicated in performance monitoring and detecting when control is necessary (MacDonald et al., 2000; Ridderinkhof et al., 2004a) possibly by conflict monitoring (Botvinick et al., 2001, 2004; Kerns et al., 2004; Kerns, 2006; Botvinick, 2007; Kim et al., 2011) or by comparing actual and predicted outcomes (Alexander and Brown, 2011), whilst lateral PFC regions including vlPFC are posited to be involved in implementing the appropriate attentional or behavioral adjustments (Ridderinkhof et al., 2004a; King et al., 2010). In one study of healthy adolescents using versions of a counting Stroop task, emotion control activated vlPFC, cognitive control activated dlPFC and both conditions activated an area in between, Brodmann Area (BA) 45 (inferior frontal gyrus), with higher activation in faster responders (Mincic, 2010). The authors suggested BA 45 serves as a common mechanism for top-down attentional control in cognitive and emotional contexts. Ochsner et al. (2008) used different versions of the Erkisen flanker task to examine response and affective conflict, observing common dACC and dlPFC activity but rostral medial PFC and left vlPFC were differentially activated by affective versus cognitive conflict. Similarly Krug and Carter (2010) used emotional and non-emotional versions of a facial Stroop task showing commonalities in dlPFC and dACC activity in both tasks.

One aspect of previous studies of top-down control of emotion that might explain discrepant findings is the nature of the emotional stimuli. Most previous research on top-down control of emotion has used emotional stimuli designed to distract from the cognitive task due to the salience of the stimulus, rather than due to any actual induced emotion. In this study we were interested in the mechanisms of emotional control that allow an individual to overcome the detrimental effects of an *experienced* emotion—induced anxiety—in order to perform a completely unrelated cognitive task.

We conducted an fMRI study of an emotional control task where anticipatory anxiety was induced by threat of shock whilst participants performed a visuospatial WM task under two load conditions (based on Shackman et al., 2006). We hypothesized that high WM load would reduce interference from threatening stimuli via an active top-down control mechanism, and that this effect would correspond to increased lateral PFC and dACC activation and decreased amygdala activation. We also aimed to test two possible active mechanisms by which interference from irrelevant stimuli can be overcome; facilitating task-related processing and inhibiting threat processing. Dynamic causal modeling was employed to compare the evidence for cognitive facilitation versus threat inhibition. We further predicted that an index of non-emotional top-down control taken from participant's performance on a dichotic listening task would predict activation in the same brain regions as emotional top-down control, pointing to a possible overlap in the neural circuitry underlying general adaptive top-down control.

## Materials and methods

### Participants

Nineteen volunteers (13 female) took part in the study. One participant was excluded from all analyses due to performing at chance on the emotional control task leaving 18 participants (13 Female) aged between 21 and 40 (mean = 25, *S.D* = 5) with normal or corrected to normal vision and hearing. All participants were right-handed and did not report any history of neurological or psychiatric problems. Participants were scanned at the University of Reading Centre for Integrative Neuroscience and Neurodynamics (CINN). Participants gave fully informed consent and the research was approved by the University of Reading Research Ethics Committee. All participants received images of their brain as compensation for their time.

### Procedure

There were two components to this study. The task used to directly assess spontaneous top-down regulation of emotion was a visuospatial WM task with threat of shock to induce anxiety. A directed dichotic listening task was used as an index of non-emotional top-down attentional control.

### Visuospatial WM task

**Figure 1:**
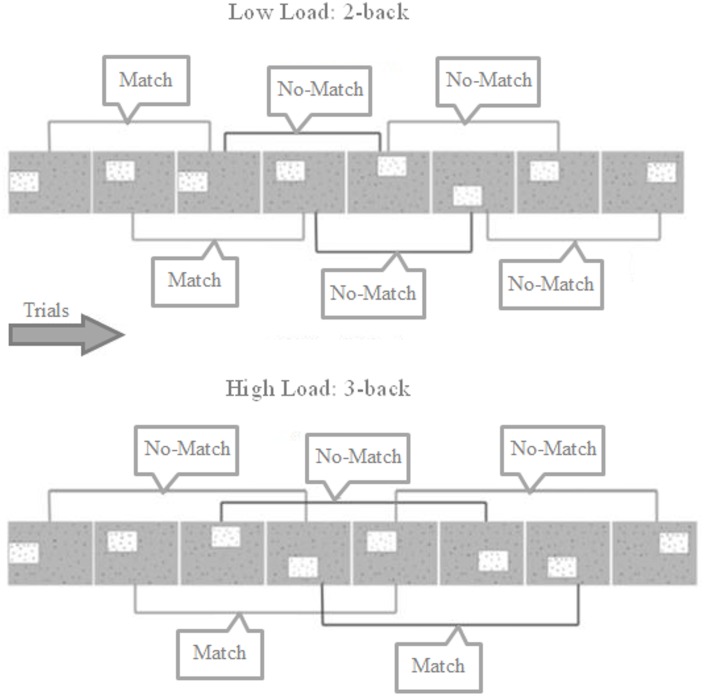
**Trial structure for 2- and 3-back blocks showing match and no-match trials.** Participants must indicate whether the box is located in the same position as 2 or 3 trials previously. Each box was presented for 350 ms, separated by 1500 ms where only the background array of random letters is shown. Participants could answer any time from the box first appearing to the next box being shown.

This n-back WM task was based on the study by Shackman et al. (2006) and consisted of a 2 (WM Load: Low Load/High Load) by 2 (Threat: Safe/Threat) within-subjects factorial design, with trials presented in blocks corresponding to the 4 experimental conditions (Low Load/Safe, Low Load/Threat, High Load/Safe and High Load/Threat. The structure of the task is shown in Figure 1. Each trial presentation consisted of a box containing one of six letters in one of eight locations; the remaining area of the display was occupied by a random array of letters. The box was presented for 350 ms, followed by an inter-trial interval (1500 ms) where the box disappeared but the background array of letters remained, following which the box reappeared. On 2-back trials participants judged whether the box displayed on the current trial occurred in the same spatial location two trials previously (and responded accordingly with a button press), whilst on 3-back trials they indicated whether the box appeared in the same location three trials previously. As in Shackman et al. (2006), the boxes appeared in overlapping, asymmetric, non-cardinal locations to encourage the use of visuospatial WM as opposed to verbal strategies. The task was presented using E-Prime 2 software (Psychological Software Tools Inc.) and a Nordic Neuro Labs goggle visual display system displaying the stimuli at 60 Hz on an 800 × 600 pixel screen, with a field of view of 30 × 23°. The goggles included a built in infrared camera for recording relative pupil dilation (recorded using 60 Hz sample rate).

The possibility of receiving an unpleasant electrical shock to the index finger of the non-dominant hand was used to induce anxiety. Blocks were either Safe or Threat as indicated before the block began and throughout by the background color (counterbalanced across participants). In Safe blocks, participants were told that there was no possibility of shock whilst in Threat blocks participants were told that they may receive one or more electric shocks. Each block began with a 4 s display indicating the type of block (2- or 3-back) with the background color representing the threat level.

During a training phase in which the visuospatial WM task was performed outside the scanner, electric shocks were delivered via an ADInstruments ML856 PowerLab 26T Isolated Stimulator using an MLADDF30 stimulating bar electrode with 30 mm spacing of 9 mm contacts. Each participant's stimulation level was set by first exposing them to an electric stimulation of 1 mA (10 pulses at 50 Hz, with a pulse duration of 200 μs) and increasing the current in steps of 0.5 mA, up to a maximum of 10 mA, until a suitable participant-specific threshold was found that was uncomfortable and unpleasant but not painful. This level was then used throughout the task for that subject (subject-specific levels ranged between 3 mA and 10 mA). Participants were told they would receive between one and 20 random shocks throughout the course of the experiment, and that the intensity of stimulation would vary. In fact, during the training WM task prior to scanning, shocks were delivered during 50% of Threat blocks. This setup allowed practice in the task, ensured that experience of the electric shock was unpleasant and that the threat of shock was capable of inducing anxiety. During the scan sham electrodes were used meaning that the scan was free from contamination by shocks, though identical instructions were given. At the end of each block, participants rated their level of anxiety in the preceding block on a sliding scale ranging from 0 (not at all anxious) to 10 (extremely anxious) moving in steps of 0.25. After the scan participants were asked whether they did in fact believe during the scan that there was a chance of receiving any electric shocks.

Each block contained 18 response trials and lasted 45 s, with 4 blocks of each of the 4 experimental conditions. As the task was repetitive in nature and required continuous concentration from the participant, the task was divided into two scan runs of eight blocks with a break period in between. The order of conditions was counterbalanced across participants.

### Dichotic listening task

The dichotic listening task (Hugdahl et al., 2009) was carried out prior to scanning on the same day as the visuospatial WM task. This involved the auditory presentation of stimuli simultaneously to the right and left ears. The stimuli consisted of six syllables comprising the stop-consonants b, d, g, p, t, and k combined with the vowel a (/ba/, /pa/, etc.). These six syllables were combined into 36 pairs (including the homonyms) with one being played to the left and the other to the right ear. Each syllable had a duration of approximately 350 ms, with an interval between presentations of 4 s. Participants repeated back the sound they heard and this was then marked down by the researcher. There were three conditions; non-forced, forced right and forced left. In the non-forced condition there was no special instruction to direct attention towards either ear, whilst in the forced right and forced left conditions participants were instructed to listen only to the right or left ear respectively and ignore any sound they heard through the other ear. Each condition composed a full run of the 36 syllable combinations. The non-forced condition was always carried out first and participants were advised not to spend too long thinking about their answer and if they believed they had heard more than one sound to indicate which sound they heard most clearly. The orders of the forced right and left conditions were counterbalanced across participants. The procedure and stimuli used have been used elsewhere (e.g., Hugdahl, 1995) and the ability to direct attention to either ear in dichotic listening tasks has been proposed as an index of top-down control (Hugdahl et al., 2009).

Participants' responses were recorded as they performed the task. These were later classified as correctly producing the syllable presented to the left ear (Correct Left), correctly producing the syllable presented to the right ear (Correct Right), or incorrect. Trials where identical sounds were presented to both ears were not scored but used to ensure typical hearing. An index of top-down control was calculated for each participant; this was taken as the sum of Correct Right in the forced right condition minus Correct Right in the non-forced condition and Correct Left in the forced left condition minus Correct Left in the non-forced condition. Higher scores represent a greater ability to direct attention to either ear compared to the control condition. The demeaned scores were used to perform a regression analysis to identify regions where activation in the emotional control task correlated with non-emotional attentional control.

### MRI acquisition

Two identical T2^*^-weighted echo planar imaging (EPI) functional scans lasting 7 min and 44 s were acquired (TR = 2 s, TE = 30 ms, flip angle = 90°, FOV = 192 × 192 mm, 3 × 3 mm voxels, slice thickness 4 mm with an interslice gap of 1 mm, 30 axial slices), separated by a short break and recalibration of the eye tracking system. Participants held an MRI-compatible response box in their dominant right hand, with the sham stimulating electrodes attached to the index finger of the left hand. Following completion of the functional scans, a high-resolution T1-weighted anatomical scan was acquired (MPRAGE, 1 × 1 mm in-plane resolution, 256 × 256 mm FOV, axial slices with 1 mm slice thickness).

### MRI data processing

fMRI analyses were carried out in Feat version 5.98 part of FSL (FMRIB's Software Library, www.fmrib.ox.ac.uk/fsl). Brain extraction was carried out using the FSL Brain Extraction Tool (BET; Smith, 2002). Motion correction using MCFLIRT (Jenkinson et al., 2002), Gaussian smoothing (FWHM 5 mm) and a 200 s high pass temporal filter were employed. First-level GLM analysis was carried out for each functional scan run and then the two runs of each participant were combined using a fixed effects analysis. Separate regressors were specified for each of the four experimental conditions (Low Load/Safe, Low Load/Threat, High Load/Safe, and High Load/Threat) by convolving a binary boxcar function with an ideal haemodynamic response. A regressor for the anxiety rating period was included, as were six motion parameters to model residual signal changes due to participant motion.

Two main effect contrasts were defined; the first to reveal WM Load-related activity by identifying regions more active in High Load compared to Low Load trials (High Load/Safe + High Load/Threat − Low Load/Safe − Low Load/Threat) and the second to reveal regions more active under threat than safety (Low Load/Threat + High Load/Threat − Low Load/Safe − High Load/Safe).

In addition to these main effect analyses, directed contrasts were set to address specific questions of this study. Firstly, a contrast was defined to identify top-down control activity by looking for areas with activation greater in the condition posited to engender top-down control (High Load/Threat) compared to all others. Secondly, a contrast to identify areas more active in the Low Load/Threat condition versus all others was defined to identify regions associated with emotional responding to threat that is reduced under high cognitive load. Given the strong a priori evidence for the role of the amygdala in negative emotions including anxiety (LeDoux, 2003; Kalin et al., 2004; Etkin and Wager, 2007; Etkin et al., 2009), a region of interest analysis was carried out with a bilateral amygdala mask (threshold 25% of the Harvard-Oxford subcortical atlas (FMRIB Software Library). Furthermore, a regression analysis with each participant's index of top-down control taken from the dichotic listening task was performed to identify how individual differences in top-down control of attention in a non-emotional task may predict individual differences in engagement of particular brain regions in the emotional control task.

Contrast images were registered to a standard space template (MNI152_T1_2 mm_brain) with FLIRT (Jenkinson and Smith, 2001; Jenkinson et al., 2002) using a two stage linear registration (functional-structural-template). Higher-level mixed effect analysis using OLS consisted of regressors for the group mean, demeaned dichotic listening scores and demeaned belief in receiving a shock (coded with 1 for expressing no doubt and −1 for expressing any). Whole-brain analysis was carried out using cluster thresholding based on Random Field Theory (Worsley, 2001) to ensure a corrected *p* < 0.05.

## Results

### Behavioral results

#### Anxiety induction

In order to reduce the effect of response biases (e.g., participants tending to cluster around one part of the scale) anxiety ratings were standardized using each participant's average rating and standard deviation across all conditions. A 2 × 2 within subject ANOVA with anxiety rating as the dependent variable revealed a significant main effect of threat of shock [*F*_(1, 17)_ = 9.697, *p* = 0.006] with a greater anxiety rating in Threat compared to Safe blocks. Neither WM Load [*F*_(1, 18)_ = 2.208, *p* = 0.155] nor the interaction [*F*_(1, 18)_ = 0.109, *p* = 0.745] reached significance. Whilst the absolute ratings of anxiety [scaled from 0 (‘not at all anxious’) to 10 (‘extremely anxious’)] were low (threat = 4.3, *S.D* = 1.33; safety = 3.6, *S.D* = 1.48), the significant main effect supports the conclusion that threat of shock successfully induced anxiety.

#### Visuospatial WM performance

In order to make decisive inferences about the differential effects of anxiety at both WM loads it is important to demonstrate psychometric equivalence. Discriminating power (Chapman and Chapman, 2001) was calculated by multiplying reliability in Safe conditions (measured by Cronbach's alpha) by the accuracy variance. This indicates the sensitivity of a test to detect an experimental manipulation. There was no significant difference in discriminating power in Low Load compared to High Load [*t*_(17)_ = −0.367, *p* = 0.718] conditions suggesting the sensitivity to detect an effect of threat was the same at both loads.

**Figure 2:**
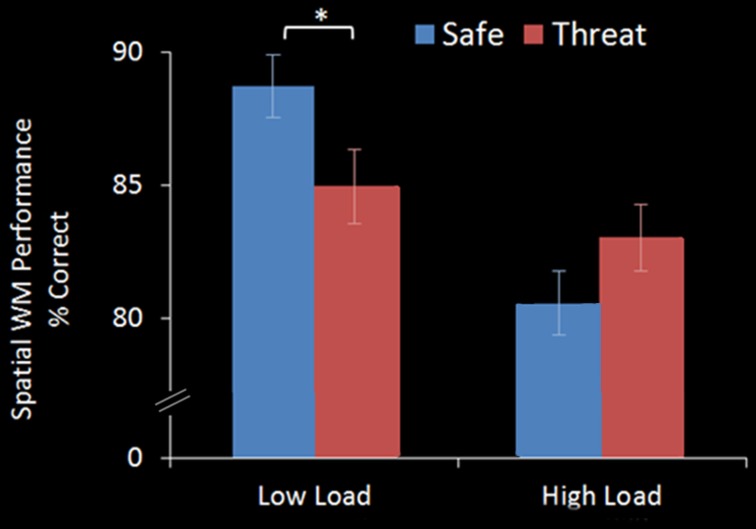
**WM accuracy in the emotional top-down control task.** In the Low Load condition (2-back) threat significantly interfered with performance, whilst in the High Load condition (3-back) there was no interference from threat. Error bars display within-subject standard error (Morey, 2008). ^*^p < 0.05 (two-tailed).

A within-subject ANOVA revealed a main effect of WM Load [*F*_(1, 17)_ = 15.865, *p* = 0.001], with significantly better performance on 2-back (86.8%, *S.D* = 10.24) than 3-back (81.8%, *S.D* = 12.21) trials. There was no main effect of Threat [*F*_(1, 17)_ = 0.422, *p* = 0.525] but a significant WM Load × Threat interaction effect [*F*_(1, 17)_ = 17.480, *p* = 0.001]. Under the Low Load condition threat of shock significantly interfered with performance [Low Load/Safe vs. Low Load/Threat; mean difference = +3.8%, *S.D* = 4.8; *t*_(17)_ = 3.370, *p* = 0.004] whereas under increased cognitive load there was no significant interference from threat of shock, in fact there was a trend for an improvement in performance [High Load/Safe vs. High Load/Threat; mean difference = −2.5%, *S.D* = 5.8; *t*_(17)_ = −1.792, *p* = 0.091] (see Figure 2).

This interference effect was not the result of a speed-accuracy trade-off; reaction times were slower in Threat (733 ms, *S.D* = 151.7) than Safe conditions (703 ms, *S.D* = 154.6) [*F*_(1, 17)_ = 14.254, *p* = 0.002] with no significant interaction effect [*F*_(1, 17)_ = 0.074, *p* = 0.789]. As expected there was a main effect of WM Load [*F*_(1, 17)_ = 4.825, *p* = 0.042] with faster responses in the 2-back (706 ms, *S.D* = 139) than 3-back (730 ms, *S.D* = 168) conditions.

### Pupil dilation

Pupil dilation has been shown to be a reliable index of cognitive effort (Beatty, 1982; Steinhauer and Hakerem, 1992) as well as reflecting emotional arousal (Bradley et al., 2008). Whilst completing the WM task in the scanner pupil width was recorded via the in-built monocular eye tracker. Due to technical issues data could only be analysed from 13 of the 19 participants. Percentage changes from the participant's mean pupil diameter were calculated for each condition and displayed a significant main effect of WM Load [*F*_(1, 14.406)_ = 10.448, *p* = 0.006] and a borderline significant WM Load × Threat interaction [*F*_(1, 18.644)_ = 4.689, *p* = 0.073]. Greatest pupil dilation was observed in the High Load/Threat condition, which drove the interaction effect. This was significantly greater than Low Load/Threat [*F*_(1, 14.864)_ = 12.429, *p* = 0.003] with no significant difference between High Load/Safe and Low Load/Safe [*F*_(1, 16.467)_ = 2.536, *p* = 0.130]. These results suggest that the greatest cognitive effort was employed under High Load/Threat conditions. Whilst pupil dilation has also been associated with emotional arousal there was no main effect of Threat [*F*_(1, 11.536)_ = 1.493, *p* = 0.130] suggesting the observed effects were not due to the additive effects of cognitive effort and arousal/pain but specifically to the increased cognitive effort in the High Load/Threat condition. This result is consistent with recent findings, for example Urry et al. (2009) demonstrated that pupil diameter increased both when increasing and decreasing an emotional response compared to maintaining it, suggesting that pupil dilation is more sensitive to modulation of cognitive demand than to small changes in emotional arousal.

### Main effect imaging results

#### Main effect of WM load: high load—low load

**Figure 3:**
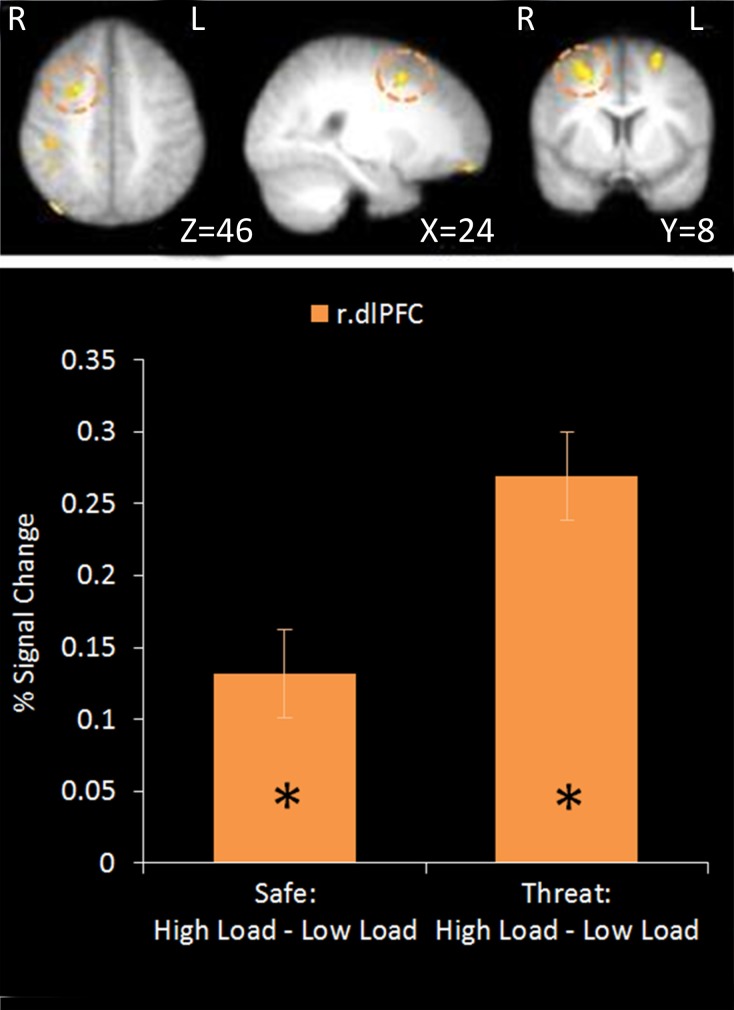
**Top: High Load—Low Load contrast imaging results.** Threshold of *z* = 2.3 uncorrected. Bottom: A right dorsolateral cluster displayed greater activation under High Load compared to Low Load in both Safe and Threat trials [High Load/Safe—Low Load/Safe: mean difference = 0.121%, *S.D* = 0.178, *t*_(17)_ = 2.880, *p* = 0.010; High Load/Threat—Low Load/Threat: mean difference = 0.162%, *S.D* = 0.188, *t*_(17)_ = 3.656, *p* = 0.002]. Error bars display within-subject standard error (Morey, 2008). ^*^p < 0.05 (two-tailed).

No regions survived whole-brain cluster corrected thresholding, however at an uncorrected z-threshold of 2.3 a cluster located in the right dlPFC was found to be significantly more active in High Load compared to Low Load blocks (see Table 1 and Figure 3). This region has been previously associated with visuospatial WM (e.g., Manoach et al., 2004), and also overlaps with frontal eye field regions suggested to be important in maintaining spatial location information during retention intervals in WM tasks (e.g., Postle, 2006; Ikkai and Curtis, 2011). There was also activation in left dlPFC as well as right parietal regions which have been implicated in working memory (Wager and Smith, 2003) and spatial cognition (Sack, 2009) and occipital regions involved in visual processing (Courtney and Ungerleider, 1997; Essen and Drury, 1997), again consistent with engagement in this spatial WM task.

**Table 1:** **Summary of imaging results for the main effect of WM Load, Threat, and the directed contrasts to investigate top-down control and emotion modulation**.

**Contrast**		**Local maxima (mm)**				**Cluster size (mm**^**3**^**)**
		***Z* score**	***x***	***y***	***z***	
High Load > Low Load Uncorrected	r. Superior frontal gyrus	3.77	24	8	46	7056
	r. Middle frontal gyrus	3.33	30	4	50	
	r. Angular gyrus	3.55	48	−50	56	5868
	r. Lateral occipital cortex, superior division	3.18	40	−64	56	
	r. Superior parietal lobule	2.63	32	−70	56	
	l. Superior frontal gyrus	3.33	−24	10	58	2988
	r. Postcentral gyrus	2.47	53	70	64	1764
Threat > Safety	Cingulate gyrus, anterior division	5.03	0	22	26	62676
	r. Frontal pole	4.67	24	58	24	
	Superior frontal gyrus	4.12	6	48	40	
	l. Inferior frontal gyrus, pars opercularis	4.78	−50	14	18	48024
	l. Inferior frontal gyrus, pars triangularis	4.39	−50	32	2	
	l. Middle frontal gyrus	4.22	−50	20	30	
	l. Frontal pole	4.21	−28	64	16	
Top-down control contrast: WM Load/Threat > Others	l. Middle frontal gyrus	3.67	−50	18	36	20952
	l. Inferior frontal gyrus, pars opercularis	3.63	−58	14	16	
	l. Inferior frontal gyrus, pars triangularis	3.57	−48	34	14	
	r. Inferior frontal gyrus, pars triangularis	4.1	54	34	12	18144
	Frontal pole	3.67	38	95	48	
	Paracingulate gyrus	3.97	−8	30	28	16740
	Cingulate gyrus, anterior division	3.61	−10	30	24	
Emotion modulation: Low Load/Threat > Others (bilateral amygdala mask)	l. Amygdala	3.24	−22	−4	−18	2268
	r. Amygdala	3.67	24	−6	−18	1836

Displaying the coordinates and Z-score of non-redundant local maxima for each cluster. Unless otherwise stated data was thresholded at z = 2.3 with cluster thresholding (Worsley, 2001) to ensure a corrected p < 0.05.

Using a mask of regions involved in WM [constrained by inference meta-analysis map based on the term “Working Memory” generated on neurosynth.org (Yarkoni et al., 2011)]. Two separate clusters were extracted from the uncorrected data; a cluster in right dlPFC and a small cluster in the angular gyrus of the right parietal cortex. Activation in the right dlPFC cluster under the High Load versus Low Load contrast displayed a positive correlation with performance on the WM task under each condition and with WM accuracy overall (*r* = 0.558, *n* = 18, *p* = 0.016). However there was no correlation with WM performance under the same WM Load contrast (*r* = 0.321, *n* = 18, *p* = 0.194). This supports the role of this region in this task and suggests that people who are able to engage this region more under High Load than Low Load conditions perform better in general on this task. The parietal cluster was equivalent to less than five voxels in native space and so further analysis was not conducted.

#### Main effect of threat: threat—safe

**Figure 4:**
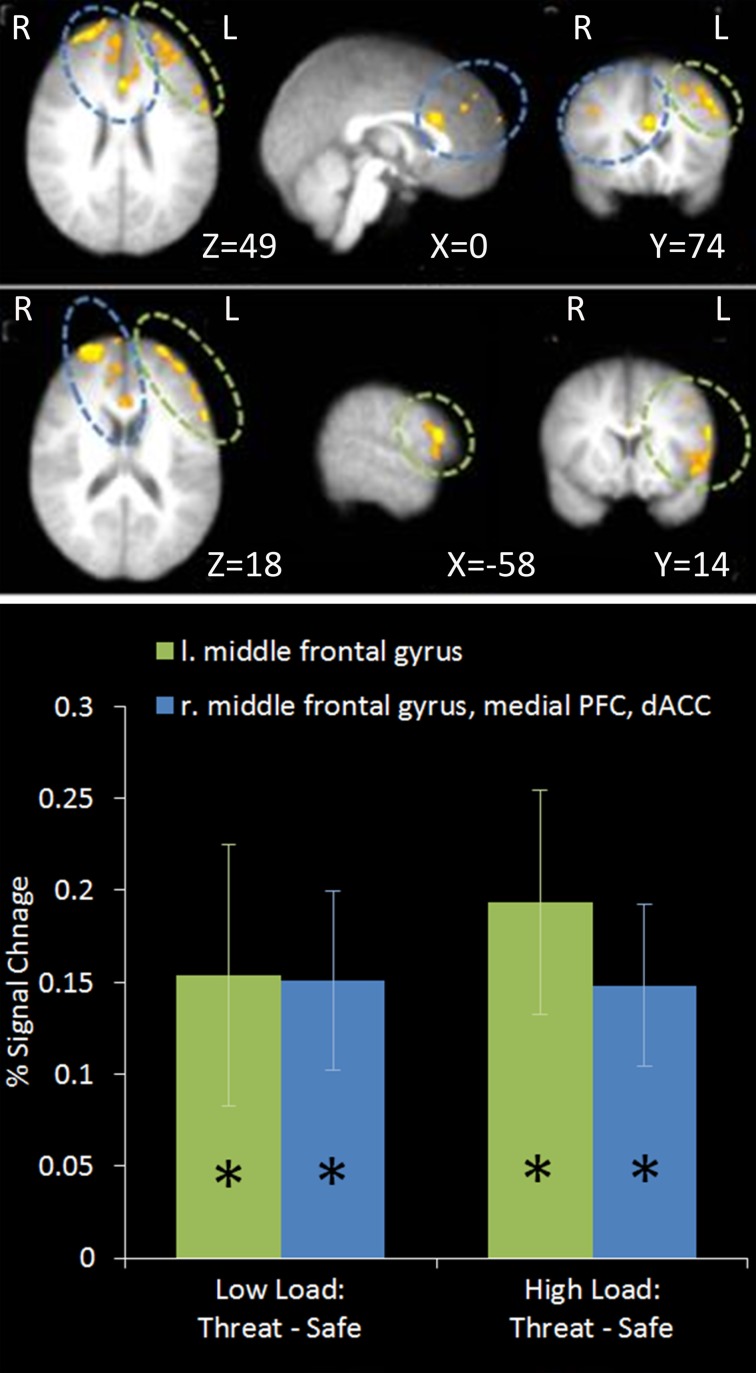
**Top: Threat—Safety contrast imaging results.** Two significant clusters were identified revealing significant bilateral middle frontal gyrus, medial PFC and dACC activation under Threat compared to Safe conditions. **Bottom:** Pairwise comparisons of the signal change in the clusters identified. Activation in both clusters was significantly greater under Threat than Safety at both Low and High Load [left middle frontal gyrus: Low Load/Threat—Low Load/Safe: mean difference = 0.153%, *S.D* = 0.213, *t*_(17)_ = 3.043, *p* = 0.007; High Load/Threat—High Load/Safe: mean difference = 0.192%, *S.D* = 0.183, *t*_(17)_ = 4.468, *p* < 0.001; right middle frontal gyrus, medial PFC and dACC cluster: Low Load/Threat—Low Load/Safe: mean difference = 0.149%, *S.D* = 0.146, *t*_(17)_ = 4.335, *p* < 0.001; High Load/Threat—High Load/Safe: mean difference = 0.147%, *S.D* = 0.132, *t*_(17)_ = 4.729, *p* < 0.001]. Error bars display within-subject standard error (Morey, 2008). ^*^p < 0.05 (two-tailed).

This contrast identified areas more active under threat of shock than safety. Such a contrast is sensitive to areas involved in anxiety but would also reveal brain regions responsible for down-regulating emotion regardless of WM Load. Two significant clusters of activation were revealed in the middle frontal gyrus bilaterally, extending from a dorsal to a more ventral lateral region in the left hemisphere, as well as the medial PFC and anterior cingulate (see Table 1 and Figure 4).

#### Top-down control contrast: high load/threat > others

**Figure 5:**
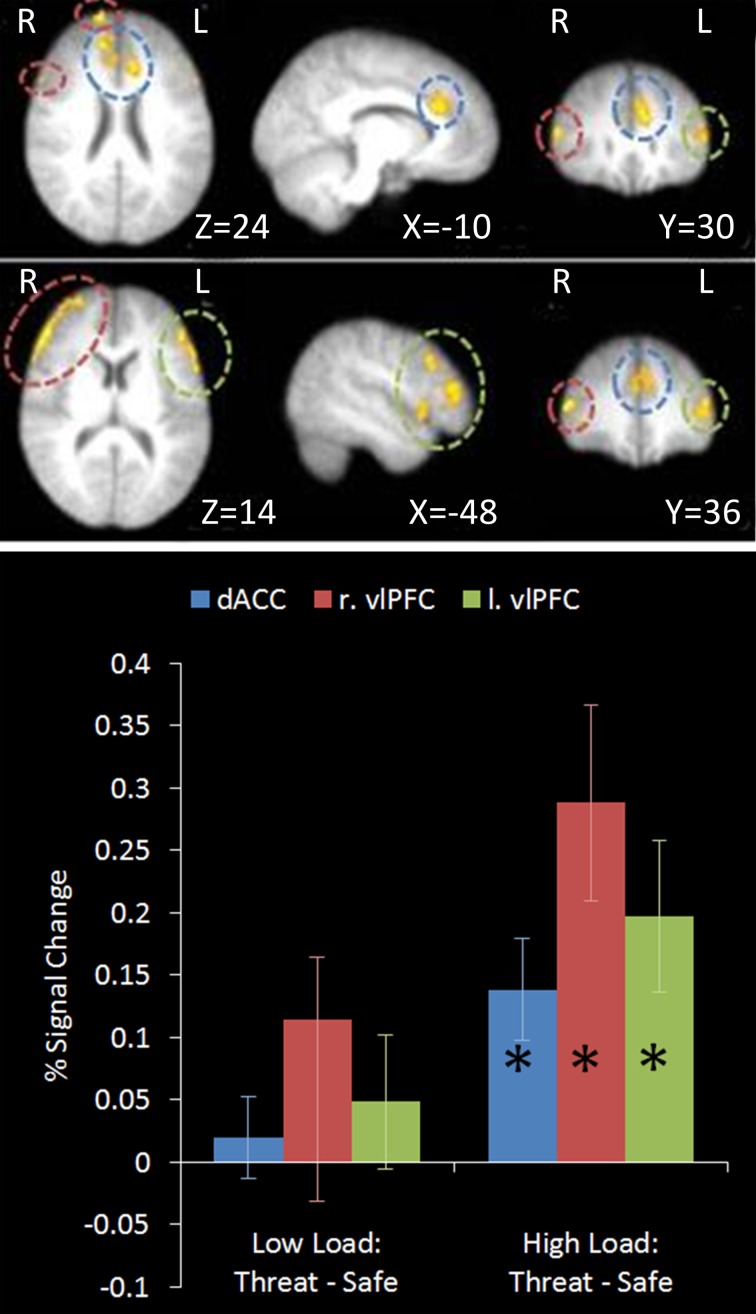
**Top: Top-down control contrast imaging results.** This contrast revealed areas where activation was greater under High Load/Threat conditions compared to all others. Three clusters were identified: Dorsal ACC as well as bilateral PFC. Medial (upper panel) and lateral (lower panel) views are displayed. **Bottom:** Pairwise comparisons of the signal change in the regions identified. Activation in all three clusters was greater under threat compared to safety under the equivalent working memory loads, however this increase was only significant under High Load [cingulate: High Load/Threat—High Load/Safe: mean difference = 0.139%, *S.D* = 0.122, *t*_(17)_ = 4.838, *p* < 0.001; left vlPFC: High Load/Threat—High Load/Safe: mean difference = 0.197%, *S.D* = 0.181, *t*_(17)_ = 4.614, *p* < 0.001; right vlPFC: High Load/Threat—High Load/Safe: mean difference = 0.288%, *S.D* = 0.236, *t*_(17)_ = 5.176, *p* < 0.001]. Error bars display within-subject standard error (Morey, 2008). ^*^p < 0.05 (two-tailed).

This contrast identified regions more active under the condition proposed to engage top-down control (High Load/Threat) compared to all others. Consistent with our hypotheses we identified a significant cluster in the anterior cingulate and paracingulate gyrus as well as bilateral vlPFC activation (see Table 1 and Figure 5). Whilst activation in these clusters under the top-down control contrast did not correlate with task performance general activation in these clusters correlated with overall task performance in both the right vlPFC and the dACC cluster (*r* = 0.629, *n* = 18, *p* = 0.005; *r* = 0.615, *n* = 18, *p* = 0.006, respectively) suggesting that participants who generally display greater recruitment of these regions whilst performing the task perform better at the task.

#### Emotion modulation contrast: low load/threat > others

**Figure 6:**
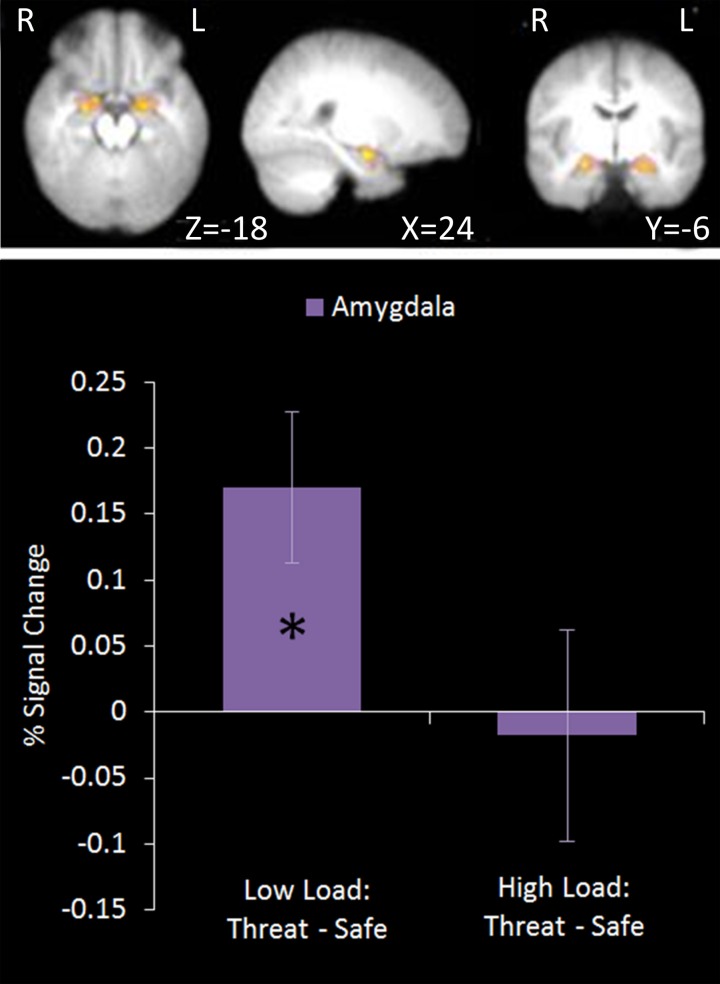
**Top: Emotion modulation contrast imaging results.** This contrast revealed areas where activation was greater under Low Load/Threat conditions compared to all others. A ROI analysis was carried out using a bilateral amygdala mask (red). **Bottom:** Pairwise comparisons of the signal change. Activation in the amygdala cluster displayed a load-dependent modulation by threat with increased amygdala activation under threat compared to safety in Low Load but not High Load conditions [Low Load/Threat—Low Load/Safe: mean difference = 0.170%, *S.D* = 0.172, *t*_(17)_ = 4.191, *p* = 0.001; High Load/Threat—High Load/Safe: mean difference = 0.018%, *S.D* = 0.254, *t*_(17)_ = 0.294, *p* < 0.772]. Error bars display within-subject standard error (Morey, 2008). ^*^p < 0.05 (two-tailed).

This region of interest analysis using an amygdala mask identified significant bilateral amygdala activation in Low Load/Threat conditions compared to all others (see Figure 6). Activation in this cluster was significantly higher under Low Load/Threat than Low Load/Safe [mean difference = 0.17%, *S.D* = 0.17; *t*_(17)_ = 4.191, *p* = 0.001] with no significant difference between High Load/Threat and High Load/Safe [mean difference = −0.02%, *S.D* = 0.25; *t*_(17)_ = 0.294, *p* = 0.772]. Activation in this cluster under this emotion modulation contrast correlated positively with overall task performance (*r* = 0.541, *n* = 18, *p* = 0.021). This correlation remained significant after controlling for Low Load/Threat—Low Load/Safe activity in this amygdala cluster (*r* = 0.518, *n* = 18, *p* = 0.033). Thus the correlation is not driven by greater amygdala reactivity to threat under Low Load, but rather suggests that general task performance in this emotional control task was related to individual differences in the load-dependent reduction of amygdala activity. However, it should be noted that activation under this contrast did not correlate with anxiety ratings (*r* = 0.130, *n* = 18, *p* = 0.606).

### Dynamic causal modelling (DCM)

Based on the proposed mechanism of top-down control of subcortical emotional regions, we employed dynamic causal modeling (DCM) (Friston et al., 2003) to probe potential connectivity in the network of regions identified in the prior analyses. All models included the right dlPFC region identified in the High Load—Low Load contrast posited to represent working memory task-related activity, left vlPFC and dACC from the top-down control contrast representing top-down control modules, and bilateral amygdala from the emotion modulation contrast. No constraints were placed on the models, permitting full bidirectional connectivity between all four nodes. Both High Load conditions provided a driving input to the right dlPFC and both Threat conditions to the amygdalae.

We were motivated to investigate the route by which top-down control may act, hypothesizing that the modulation of the detrimental effect of induced anxiety could proceed by inhibiting some level of emotion processing, by facilitating task related activity, or some combination of the two. To test this, we used family inference, whereby families of models that differ only on specific features of interest are contrasted in order to provide evidence for or against this specific characteristic (Penny et al., 2010). In such inference, individual models in each family are modeled as random samples from the total possible model space, in much the same way as participants in an experiment are modeled as random samples from a population. Accordingly, we constructed three families with the same basic architecture described above but differing in the target of top-down control: Emotion Modulation—30 models representing inhibition of emotion processing where all possible combinations of connections between the amygdala and the two top-down nodes were modulated by either High Load/Threat conditions or dACC activation, Working Memory Modulation—30 models representing facilitation of task processing where all possible combinations of connections between the right dlPFC and the top-down control nodes were modulated, and Combined—30 models representing both emotional control and task-related control constrained such that in each model the equivalent connections between the top-down nodes and emotion and task nodes were modulated, resulting in the same number of models in each family.

**Figure 7:**
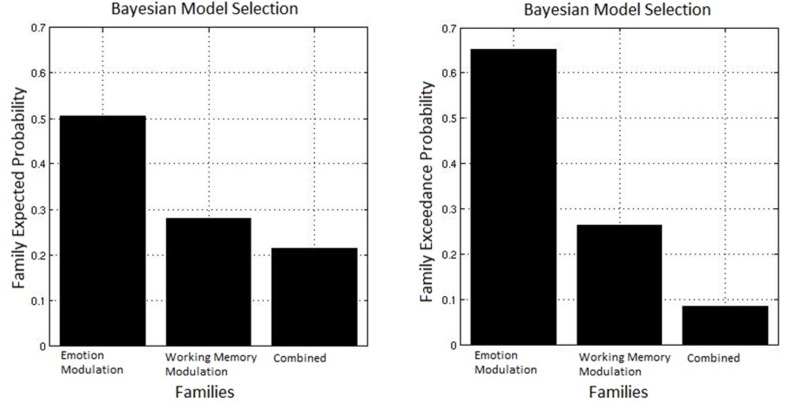
**Bayesian model selection for families.** The Emotion Modulation family displayed the greatest exceedance probability, favoring models where connections between top-down nodes and the amygdala were modulated over ones with modulation of connections between top-down and task-related nodes.

The observed data were fit to each model, and random effect Bayesian model selection was used to estimate the posterior probabilities of each model given the data. A random effects analysis was used to permit different participants to favor different models as may be the case if individual differences determine the predominance of an inhibitory or facilitative route. Based on this the exceedance probability can be computed for each family, this represents the probability that one family is more likely than any other given the group data. Figure 7 displays the exceedance probabilities for each family; this identified the winning family as Emotion Modulation; the exceedance probability for this family was 0.653 meaning we can be 65.3% confident that it has a greater posterior probability than any other family. The exceedance probability for the Working Memory Modulation family was 0.263 and 0.085 for Combined (though it must be noted that this family had the arbitrary constraint of only including equivalent modulations of Emotion and Working Memory connections). This result favors inhibition of emotional processing as the most probable route by which top-down control acts in this specific task.

**Figure 8:**
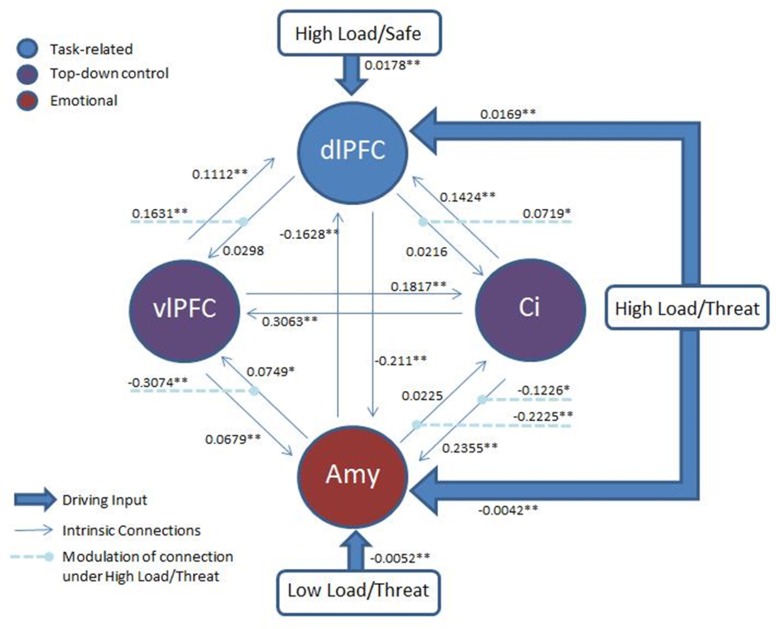
**Architecture of the model: Two top-down control nodes, left vlPFC and cingulate, from the top-down control contrast, a task-related node, right dlPFC, from the uncorrected High Load—Low Load contrast and an emotion node from the emotion modulation contrast.** All threatening conditions had a driving input to the amygdala and all High Load conditions to the right dlPFC. Modulation of connections by High Load/Threat are also displayed. Full connectivity was permitted between all nodes. Average parameter estimates for each intrinsic connection, driving input and modulation are displayed. ^*^p < 0.05 (two-tailed; uncorrected). ^**^p < 0.05(two-tailed, Bonferonni correction applied).

Classical inference on the parameter estimates across participants (weighted by the evidence of each model for each participant) was conducted with a Bonferonni corrected p-threshold of 0.0018 (see Figure 8 for model architecture and parameter estimates). This revealed a significant decrease in both the amygdalae to dACC connection [*t*_(17)_ = −6.273, *p* = 0.00009] and amygdalae to vlPFC connection [*t*_(17)_ = −6.297, *p* < 0.00008] under High Load/Threat conditions. The modulation of the connection from the dACC to amygdalae displayed only a trend to decrease [*t*_(17)_ = −2.449, *p* = 0.025] and the vlPFC to amygdalae connection did not display significant modulation under High Load/Threat conditions [*t*_(17)_ = −0.855, *p* = 0.404]. Additionally, the dlPFC to vlPFC connections were significantly increased by High Load/Threat [*t*_(17)_ = 5.351, *p* = 0.000053] whilst the dlPFC to dACC connection displayed a borderline significant modulation[*t*_(17)_ = 3.168, *p* = 0.0056].

Taken together these results suggest that whilst the family inference favors inhibition of emotion, there is also some evidence (in the dlPFC to vlPFC connection) for facilitation of WM-related activity. Furthermore, there is stronger evidence that this mechanism acts by suppressing the output of the amygdala in the case of inhibition of emotional processing and by boosting the output of the dlPFC in the case of facilitating task activity than by modulating the activity of these regions directly. Additionally, we found significant negative bidirectional connectivity between the amygdala and right dlPFC [amygdala to dlPFC: *t*_(17)_ = −7.573, *p* = 0.000001; dlPFC to amygdala: *t*_(17)_ = −11.543, *p* < 0.000001], consistent with interacting inhibitory emotional and cognitive networks (Dolcos and McCarthy, 2006; Dolcos et al., 2008; Dichter et al., 2010). Given that amygdala and dlPFC have few if any direct structural connections (Porrino et al., 1981; Barbas and De Olmos, 1990; Ghashghaei and Barbas, 2002; Freese and Amaral, 2009), this last result suggests that a mutually inhibitory cognitive-emotional connection cannot be adequately explained by indirect pathways through the vlPFC or dACC, implying the existence of another indirect pathway.

### Regression analysis of non-emotional top-down control scores

We hypothesized that individual differences in people's performance on a non-emotional top-down control task would predict their recruitment of regions implicated in top-down control of emotion. To address this issue a regression analysis was performed using the index of non-emotional top-down control taken from the behavioral dichotic listening task as a regressor in the between subjects GLM of the emotional top-down control contrast. This would reveal brain regions for which non-emotional top-down control ability predicts activation associated with emotional top-down control.

**Figure 9:**
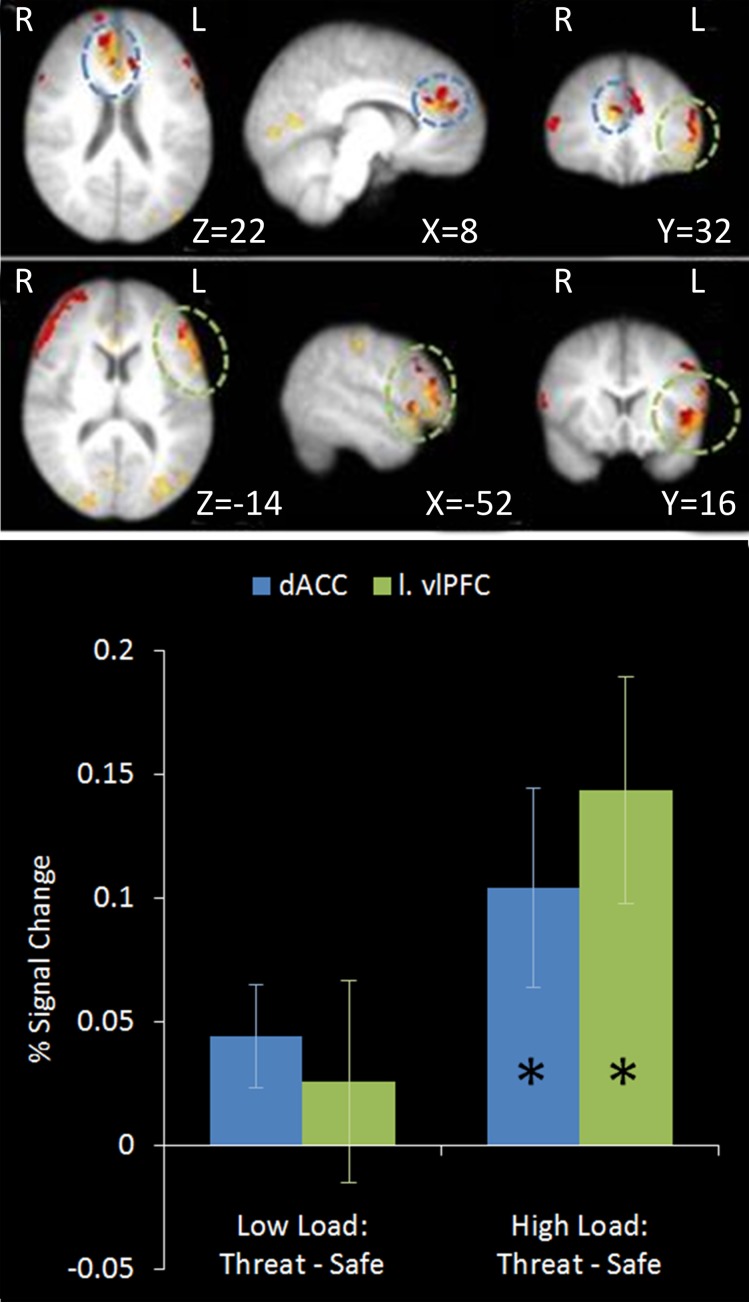
**Top: Top-down control regression imaging results overlaid on main effect top-down control (red).** This identified regions where activation under the top-down control contrast in the emotional control task correlated with the index of attentional control from the dichotic listening task. Medial (upper panel) and lateral (lower panel) views are displayed. Dorsal ACC and left vlPFC identified by this analysis displayed some overlap with the main effect top-down control results. Bottom: Pairwise comparisons of the signal change in the regions identified. Both regions displayed a significant increase in activation under threat compared to safety in High Load conditions [Cingulate: High Load/Threat—High Load/Safe: mean difference = 0.104%, *S.D* = 0.170, *t*_(17)_ = 2.585, *p* = 0.019; left vlPFC: High Load/Threat—High Load/Safe: mean difference = 0.143%, *S.D* = 0.194, *t*_(17)_ = 3.133, *p* = 0.006]. The cingulate also displayed a borderline significant increase under threat in Low Load conditions [mean difference = 0.044%, *S.D* = 0.089, *t*_(17)_ = 2.094, *p* = 0.052]. Error bars display within-subject standard error (Morey, 2008). ^*^p < 0.05 (two-tailed).

A number of regions displayed this relationship (see Table 2). As hypothesized both the left vlPFC and dACC showed greater activation under this top-down control contrast in subjects who were better at the unrelated non-emotional attentional control task (left vlPFC: *r* = 0.927, *n* = 18, *p* < 0.001; cingulate: *r* = 0.825, *n* = 18, *p* < 0.001) (see Figure 9).

**Table 2:** **Summary of imaging results for regression analysis under the top-down control contrast**.

**Contrast**		**Local maxima (mm)**				**Cluster size (mm**^**3**^**)**	**Pearson's r. *N* = 18**
		***Z* score**	***x***	***y***	***z***		
Dichotic listening: Top-down control contrast regression analysis	Visual cortex V1	4.46	2	−80	12	50220	0.843^**^
	r. Lateral occipital cortex, inferior division	4.07	30	−88	0		
	l. Inferior frontal gyrus, pars opercularis	4.25	−54	16	0	33048	0.928^**^
	l. Inferior frontal gyrus, pars triangularis	3.74	−52	26	14		
	l. Precentral gyrus	4.42	−42	−16	56	22932	0.606^**^
	l. Postcentral gyrus	3.9	−42	−34	56		
	r. Inferior parietal lobule	3.5	60	−30	44	15012	0.709^**^
	r. Postcentral gyrus	3.41	36	−30	48		
	Paracingulate gyrus	4.11	4	48	20	13536	0.828^**^
	Cingulate gyrus, anterior division	3.69	0	22	22		
	l. Lateral occipital cortex, superior division	4.01	−34	−80	18	13212	0.714^**^
	l. Lateral occipital cortex, inferior division	3.41	−46	−78	−6		

*This identifies regions whose activation under the top-down control contrast (High Load/Threat > Others) correlates with dichotic listening scores; Pearson's r are displayed*.

** indicates significance at 01 (two-tailed). Data were thresholded at z = 2.3 with cluster thresholding (Worsley, 2001) to ensure a corrected p < 0.05.

In addition to prefrontal regions implicated in top-down control widespread activation related to visual processing was discovered by this analysis, including a large cluster covering right lateral and mid occipital cortex, V1 and V2 as well as a smaller cluster in left lateral occipital cortex. This finding was not hypothesized but is consistent with individual differences in the ability to direct attention in the dichotic listening correlating with the ability to direct attention to the visual domain in this spatial WM task. Clusters were also identified bilaterally in the postcentral gyrus of the parietal cortex, relating to primary somatosensory cortex.

Activity in these regions specifically under the top-down control contrast correlated with performance on the dichotic listening task; general activity in these regions did not (with the exception of the left somatosensory cluster (*r* = −0.548, *n* = 18, *p* = 0.019). Thus the index of top-down attentional control predicts the recruitment of these regions under the conditions posited to require top-down emotional control, and not their general recruitment across all conditions. Furthermore, there was no direct correlation between the index of top-down control on the dichotic listening task and WM performance on the emotional control task (*r* = 0.191, *n* = 18, *p* = 0.447).

In the emotional control task, error rates differed across conditions and so present a potential confound for the results since dACC has been shown to be sensitive to errors (Kiehl et al., 2000; Menon et al., 2001). However activity under the top-down control contrast in both the cingulate cluster identified in the main effect analysis and in the regression analysis did not show any correlation with errors made in the WM task (*r* = 0.366, *n* = 18, *p* = 0.135; *r* = 0.239, *n* = 18, *p* = 0.341), meaning that activity observed in this contrast cannot be attributed to higher error rates.

## Discussion

The current study investigated how interfering effects of threat induced anxiety can be modulated in order to focus on current tasks. We demonstrated how threat-related interference can be overcome by increasing the load of a cognitive task; in this case interference of anxiety under threat of shock on a visual spatial WM task was eradicated when the WM load was increased. Activation in dorsal ACC and ventrolateral PFC under high working memory load with threat of shock was consistent with their hypothesized roles in top-down control, suggesting they are recruited to modulate the interfering effects of emotion. Furthermore, a bilateral amygdala cluster displayed significantly greater activation under threat of shock compared to safety at low load but no effect of threat at a higher WM load, providing evidence that cognitive load can modulate threat-related amygdala activity. Dynamical Causal Modeling further suggested that this top-down control might be achieved through inhibition of ascending outputs from the amygdala to the prefrontal cortex.

The interaction between anxiety and cognition is not straightforward; anxiety is an adaptive mechanism that plays a vital role in warning of potential threats which might occur at any time, including when we are occupied with other activities. Accordingly, anxiety increases sensitivity to potential threats even when people are engaged in highly demanding perceptual tasks (Cornwell et al., 2007, 2011). Anxiety can disrupt ongoing cognitive processing due to competition for limited capacity WM resources (e.g., Lavie et al., 2004), for visuospatial attention resources (Shackman et al., 2006), by disrupting the functioning of the goal-directed attentional system (Eysenck et al., 2007), or via an automatic reciprocal interaction between ventral ‘limbic’ regions and dorsal executive areas (e.g., Dolcos and McCarthy, 2006; Dolcos et al., 2008; Dichter et al., 2010). Despite its privileged role in alerting us of danger, however, it is clear that interference by anxiety can be overcome in certain situations. Our results are consistent with a number of studies that suggest that increasing cognitive or attentional load reduces processing in emotion response regions such as the amygdala. For example, Taylor et al. (2003) and Northoff et al. (2004) found that even relatively simple tasks can affect neural activation in emotion-associated regions, and Van Dillen et al. (2009) demonstrated that activation in the amygdala is reduced by increasing cognitive load even when the emotional stimuli precede the task.

These results highlight a potential difference between top-down control of emotional versus non-emotional interference. In the non-emotional domain, for example, evidence exists that when cognitive/executive load is increased, interference from task-irrelevant information is exacerbated, as explained by the load theory of attention and cognitive control proposed by Lavie et al. (2004). It is possible, however, that at least partially separate mechanisms exist for the top-down control of emotion. Emotional stimuli are a special case of distractors; although they might be irrelevant to the explicit task, they signal events with high potential significance to wellbeing and are preferentially and automatically processed (e.g., Dolan and Vuilleumier, 2003; Alpers and Gerdes, 2007; Stout et al., 2013). This might particularly be the case when the interference comes from an induced emotion as opposed to perception of an emotional stimulus (which may or may not elicit an emotional response). If emotional information can be automatically processed, there is no reason why it should necessarily interfere with cognitive processing—whether or not it does so might therefore be dependent on the nature of the ongoing task and the strength of top-down control. Although Shackman et al. (2006), observed interference from threat of shock in a 3-back condition, we only observed this interference at a lower WM load (2-back) using a very similar task. However, differences in task difficulty might explain this apparent discrepancy; Shackman et al. (2006) employed 6 different locations with the stimuli presented for 500 ms with 2500 ms intervals between presentations. In contrast, the current study, based on piloting evidence, employed 8 locations [differing in position and extent of overlap to Shackman et al. (2006)] and the stimuli were present for just 350 ms with 1500 ms intervals. Future psychophysical studies will be needed to characterize under exactly what WM load or task difficulty conditions top-down control reduces threat-related interference.

If threat cannot be processed entirely automatically, however, an alternative explanation to an active top-down control theory that could account for the reduced interference of threat under greater cognitive task demands must be acknowledged. Depletion of cognitive or attentional resources might prevent emotional stimuli from being processed sufficiently to interfere with the task (the latter case would be similar to how Lavie et al. (2004) explain the lack of interference from perceptual distractors under high perceptual load). In this experiment, we used threat of shock, rather than shock itself or other unpleasant stimuli such as pictures presented concurrent with the WM task, so that there was no overt emotional stimulus to be processed during task performance. However, it is still possible that emotional information such as heightened anxiety, even in the absence of a stimulus, might not be processed under high WM load due to WM capacity limits. Despite evidence from a large number of studies suggesting that affective stimuli can be at least partially processed automatically without the need for attention (e.g., Morris et al., 1998, 2001; Dolan and Vuilleumier, 2003), some studies suggest that this is not always the case (e.g., Pessoa et al., 2002). However, although the finding of a load dependent modulation of amygdala activity in the current study is consistent with both an active control mechanism and a resource depletion account, regions implicated in top-down control were recruited under High Load/Threat conditions and a Threat × WM Load interaction was observed in the pupil data, with greatest dilation under High Load/Threat. Both these results suggest that there was increased cognitive load in the High Load/Threat condition (Beatty, 1982; Steinhauer and Hakerem, 1992; Johnstone et al., 2007), which would not be expected if threat information was not being processed. It seems likely, then, that in the high cognitive load condition, threat information was processed but was actively prevented from interfering with WM performance.

The proposed active control mechanism could act by facilitating the task at hand, inhibiting the interfering effect of the emotion or a combination of the two. Some studies suggest there are dissociable neural systems implementing top-down control in emotional and non-emotional contexts (Ochsner et al., 2008; Mincic, 2010), with the distinction being that non-emotional interference is overcome with facilitation of task-related activity whilst emotional interference is overcome by active inhibition (Egner et al., 2008). In the current study there was greater support for the active inhibition of emotional processing; amygdala activation under Threat displayed a load-dependent modulation, with no activation to threat of shock at High Load. Under the High Load condition, however, there was also a trend for improvement in WM accuracy under Threat compared to Safe conditions, which might suggest some role of facilitation of task performance under High Load and Threat. Additionally, the increased cognitive effort in High Load/Threat trials indicated by the pupil dilation data may represent either the additional engagement of top-down regulatory mechanisms of the emotional interference or increased cognitive effort in the WM task.

Dynamic causal modeling was exploited to further probe the proposed circuitry. Models were partitioned into distinct families dependent on whether the pattern of modulation of connections was consistent with an emotion inhibition or WM task facilitation account. Family level inference found greater evidence that emotional interference is overcome by inhibition of emotional processing. Interestingly, the DCM analysis provided evidence that top-down control acts via the modulation of amygdala output, in addition to direct reduction of amygdala activity. The current results relate only to estimates of effective connectivity. Determining the precise anatomical routes and physiological mechanisms by which this control is implemented is a challenge for future studies, perhaps making use of diffusion tensor imaging to characterize the white matter pathways and pharmacological manipulations and/or magnetic resonance spectroscopy to understand the neurotransmitters involved.

A related issue is that although the ability to modulate interfering effects of emotion is significant in itself, it is not clear whether this requires a concomitant regulation in emotional experience. This highlights the multifactorial nature of emotions, which are made up of subjective feelings, physiological and neurological responses, as well as cognitive processes and action tendencies (Scherer, 2000). Whilst these components are related, their interaction with ongoing cognitive task demands may be somewhat distinct. For example, Dvorak-Bertsch et al. (2007) demonstrated that fear-potentiated startle can be modulated by working memory load and Vytal et al. (2012) observed eradication of interference from anxiety and diminished fear-potentiated startle with increasing WM load. In contrast, the current study observed a load-dependent modulation of emotional interference in the WM task along with a load-dependent modulation of amygdala activation, but failed to regulate subjective ratings of anxiety. This may reflect the relative lack of sensitivity of the self-report measure and/or demand characteristics, or that the top-down control required to focus on the task does not reduce the subjective experience of anxiety. It should be noted that the current study is limited by the lack of additional valence sensitive online measures of emotion, such as facial EMG, or skin conductance measures.

We also examined whether individual differences in the performance of a completely non-emotional attentional control task (a dichotic listening task) would predict the recruitment of brain regions involved in the top-down control of threat. A regression analysis identified a number of such brain regions including both dACC and left vlPFC. Dorsal ACC and vlPFC have been associated with both emotion regulation (Blair et al., 2007; Ochsner and Gross, 2008; Van Dillen et al., 2009) and cognitive control (Herath et al., 2001; Dux et al., 2006). We propose that this correlation reflects these brain regions' common roles in both top-down control during a WM task in the presence of threat-provoked anxiety as well as during an auditory task with the need to selectively ignore irrelevant, non-emotional auditory information. The two tasks, as well as the type of distracting information, were deliberately chosen to be very different, making it unlikely that findings common to both tasks are due to the specifics of the stimuli or of task-specific processing demands.

Several studies have implicated regions of the cingulate in different types of cognitive control (see Vogt et al., 1992; Carter et al., 1999; Bush et al., 2000; Shackman et al., 2011 for reviews). Specifically, studies posit a role in monitoring when top-down control is required and recruiting the appropriate regions to implement this control (Ridderinkhof et al., 2004a,b). Many studies suggest the cingulate monitors conflict including studies of the Stroop task (Kerns et al., 2004), Simon task (Peterson et al., 2002; Kerns, 2006), and go/no-go paradigms (Braver et al., 2001), with evidence suggesting this conflict indicates the need for top-down control. Detection of conflict by the cingulate leads to recruitment of prefrontal regions necessary to implement this control (Kerns et al., 2004; Kerns, 2006). Similar regions of ACC have also been implicated in the processing of pain and affect. A recent meta-analysis of 192 imaging studies of cognitive control, negative affect and pain (Shackman et al., 2011) identified a region of the cingulate largely overlapping the region we found in the regression on dichotic listening scores (see Figure 9). Shackman et al. (2011) propose that this region of cingulate serves a general role in adaptive control, defined as being ‘to bias responding in situations where the optimal course of action is uncertain or entails competition between alternative courses’ (Shackman et al., 2011, p. 161). The connections of this region of cingulate cortex with other brain regions would support different types of adaptive control depending on the specific context. Connections with pre-motor and motor regions make dACC a suitable candidate for modifying, initiating or potentiating task-relevant motor actions. Connections with dorsal and lateral prefrontal cortex would enable biasing of attention and WM resources towards task-relevant information while connections with ventral prefrontal areas and limbic regions would allow for direct modulation of emotional responses. Thus whilst the dACC may serve an important role in emotion regulation, viewing its function in a broader context could help to elucidate more fully the role it plays. Whilst the current results are consistent with this domain general view of dACC function the conclusions that can be drawn from such regression analyses are limited and the correlation between recruitment of these regions and dichotic listening performance could be mediated by other factors. In order to fully address this it is necessary to perform functional imaging on the same participants completing both emotional and non-emotional top-down control tasks in future studies.

A further point is that although we hypothesize the existence of domain-general top-down control mechanisms which can be recruited in different contexts, they do not preclude the existence of neural circuitry involved in emotion regulation more specifically. For example, in the case of more voluntary emotion regulation such as that engaged in studies of emotion reappraisal, there is substantial evidence for the involvement of neural regions such as the orbitofrontal cortex (Lévesque et al., 2003; Goldin et al., 2008) that assign, or reassign, affective meaning or hedonic value to stimuli. In such situations then, one might expect domain-general mechanisms to interact with more emotion-specific or process-specific mechanisms.

The precise mechanisms by which top-down control can maintain task performance in the presence of threat or other sources of emotional interference, and the conditions under which such control mechanisms are effective or break down is highly clinically relevant. For example dysfunction in the neural circuitry that supports the top-down regulation of emotion has been demonstrated in several psychological disorders including bipolar disorder (Foland et al., 2008), schizophrenia (Blasi et al., 2009), depression (Johnstone et al., 2007; Joormann et al., 2007), PTSD (Shin et al., 2001) and several anxiety disorders (Campbell-Sills et al., 2011; Blair et al., 2012). Indeed, the wide range of psychopathologies linked to deficits in emotion regulation has been recognized in several recent reviews (Phillips et al., 2003; Taylor and Liberzon, 2007; Amstadter, 2008; Dillon et al., 2011; Berking and Wupperman, 2012). Studies of these psychopathologies have understandably focused on the affective nature of any deficits, however a relationship between emotional cognitive control and non-affective cognitive control suggests that deficits in other domains may also be apparent. For example, trait anxiety is linked to a diminished recruitment of prefrontal attentional control mechanisms to inhibit the processing of non-emotional distractors (Bishop, 2009) and deficits in executive functions have been observed in a number of psychological disorders including depression (Paelecke-Habermann et al., 2005; Siegle et al., 2007), obsessive-compulsive disorder, and schizophrenia (Moritz et al., 2002). It is possible that specific combinations of dysfunction in emotion-specific versus domain-general adaptive control will correspond to distinct symptoms or sub-categories of mood and affective disorders, though this speculative proposal has yet to be tested.

The current study demonstrates a load-dependent modulation of the interfering effects of induced anxiety and provides evidence that this occurs by an active mechanism favoring inhibition of emotional processing over task facilitation, though the predominance of either route under different contexts requires further study. We also show that the ability to exert attentional control on a completely independent non-emotional task predicts the recruitment of vlPFC and dACC in this emotional control task, consistent with a proposed role in domain general top-down control, of which emotion regulation is just one example. The interaction between these emotional and cognitive networks is relevant to the understanding of a range of psychopathologies and further elucidation of how these networks interact as well as how they are modulated under different contexts is crucial.

### Conflict of interest statement

The authors declare that the research was conducted in the absence of any commercial or financial relationships that could be construed as a potential conflict of interest.

## References

[B1] Alexander W. H. Brown J. W. (2011). Medial prefrontal cortex as an action-outcome predictor. Nat. Neurosci. 14, 1338–1344 10.1038/nn.2921 21926982 PMC3183374

[B2] Alpers G. W. Gerdes A. B. (2007). Here is looking at you: emotional faces predominate in binocular rivalry. Emotion 7, 495–506 10.1037/1528-3542.7.3.495 17683206

[B3] Amstadter A. B. (2008). Emotion regulation and anxiety disorders. J. Anxiety Disord. 22, 211 10.1016/j.janxdis.2007.02.004 17349775 PMC2736046

[B4] Barbas H. De Olmos J. (1990). Projections from the amygdala to basoventral and mediodorsal prefrontal regions in the rhesus monkey. J. Comp. Neurol. 300, 549–571 10.1002/cne.903000409 2273093

[B5] Beatty J. (1982). Task-evoked pupillary responses, processing load, and the structure of processing resources. Psychol. Bull. 91, 276 10.1037/0033-2909.91.2.276 7071262

[B6] Berking M. Wupperman P. (2012). Emotion regulation and mental health. Curr. Opin. Psychiatry 25, 128–134 10.1097/YCO.0b013e3283503669 22262030

[B7] Bishop S. Duncan J. Brett M. Lawrence A. D. (2004). Prefrontal cortical function and anxiety: controlling attention to threat-related stimuli. Nat. Neurosci. 7, 184–188 10.1038/nn1173 14703573

[B8] Bishop S. J. (2009). Trait anxiety and impoverished prefrontal control of attention. Nat. Neurosci. 12, 92–98 10.1038/nn.2242 19079249

[B9] Blair K. S. Smith B. W. Mitchell D. G. V. Morton J. Vythilingam M. Pessoa L. (2007). Modulation of emotion by cognition and cognition by emotion. Neuroimage 35, 430–440 10.1016/j.neuroimage.2006.11.048 17239620 PMC1862681

[B10] Blair K. S. Geraci M. Smith B. W. Hollon N. DeVido J. Otero M. (2012). Reduced dorsal anterior cingulate cortical activity during emotional regulation and top-down attentional control in generalized social phobia, generalized anxiety disorder, and comorbid generalized social phobia/generalized anxiety disorder. Biol. Psychiatry 72, 476–482 10.1016/j.biopsych.2012.04.013 22592057 PMC3424322

[B11] Blasi G. Popolizio T. Taurisano P. Caforio G. Romano R. Di Giorgio A. (2009). Changes in prefrontal and amygdala activity during olanzapine treatment in schizophrenia. Psychiatry Res. 173, 31–38 10.1016/j.pscychresns.2008.09.001 19428222 PMC2736305

[B12a] Botvinick M. M. (2007). Conflict monitoring and decision making: reconciling two perspectives on anterior cingulate function. Cogn. Affect. Behav. Neurosci. 7, 356–366 18189009 10.3758/cabn.7.4.356

[B12] Botvinick M. M. Braver T. S. Barch D. M. Carter C. S. Cohen J. D. (2001). Conflict monitoring and cognitive control. Psychol. Rev. 108, 624–652 10.1037/0033-295X.108.3.624 11488380

[B13] Botvinick M. M. Cohen J. D. Carter C. S. (2004). Conflict monitoring and anterior cingulate cortex: an update. Trends Cogn. Sci. 8, 539–546 10.1016/j.tics.2004.10.003 15556023

[B13a] Bradley M. M. Miccoli L. Escrig M. A. Lang P. J. (2008). The pupil as a measure of emotional arousal and autonomic activation. Psychophysiol. 45, 602–607 10.1111/j.1469-8986.2008.00654.x 18282202 PMC3612940

[B14] Braver T. S. Barch D. M. Gray J. R. Molfese D. L. Snyder A. (2001). Anterior cingulate cortex and response conflict: effects of frequency, inhibition and errors. Cereb. Cortex 11, 825–836 10.1093/cercor/11.9.825 11532888

[B15] Bush G. Luu P. Posner M. I. (2000). Cognitive and emotional influences in anterior cingulate cortex. Trends Cogn. Sci. 4, 215–222 10.1016/S1364-6613(00)01483-2 10827444

[B16] Campbell-Sills L. Simmons A. N. Lovero K. L. Rochlin A. A. Paulus M. P. Stein M. B. (2011). Functioning of neural systems supporting emotion regulation in anxiety-prone individuals. Neuroimage 54, 689–696 10.1016/j.neuroimage.2010.07.041 20673804 PMC2962684

[B17] Carter C. S. Botvinick M. M. Cohen J. D. (1999). The contribution of the anterior cingulate cortex to executive processes in cognition. Rev. Neurosci. 10, 49–57 10.1515/REVNEURO.1999.10.1.49 10356991

[B18] Chapman L. J. Chapman J. P. (2001). Commentary on two articles concerning generalized and specific cognitive deficits. J. Abnorm. Psychol. 110, 31–39 10.1037/0021-843X.110.1.31 11261396

[B19] Cisler J. M. Koster E. H. W. (2010). Mechanisms of attentional biases towards threat in the anxiety disorders: an integrative review. Clin. Psychol. Rev. 30, 203 10.1016/j.cpr.2009.11.003 20005616 PMC2814889

[B20] Cornwell B. R. Baas J. M. P. Johnson L. Holroyd T. Carver F. W. Lissek S. (2007). Neural responses to auditory stimulus deviance under threat of electric shock revealed by spatially-filtered magnetoencephalography. Neuroimage 37, 282 10.1016/j.neuroimage.2007.04.055 17566766 PMC2717627

[B21] Cornwell B. R. Alvarez R. P. Lissek S. Kaplan R. Ernst M. Grillon C. (2011). Anxiety overrides the blocking effects of high perceptual load on amygdala reactivity to threat-related distractors. Neuropsychologia 49, 1363–1368 10.1016/j.neuropsychologia.2011.02.049 21376745 PMC3085886

[B22] Courtney S. M. Ungerleider L. G. (1997). What fMRI has taught us about human vision. Curr. Opin. Neurobiol. 7, 554–561 10.1016/S0959-4388(97)80036-0 9287197

[B24] Dichter G. S. Bellion C. Casp M. Belger A. (2010). Impaired modulation of attention and emotion in schizophrenia. Schizophr. Bull. 36, 595–606 10.1093/schbul/sbn118 18843096 PMC2879691

[B25] Dillon D. G. Deveney C. M. Pizzagalli D. A. (2011). From basic processes to real-world problems: how research on emotion and emotion regulation can inform understanding of psychopathology, and vice versa. Emotion Rev. 3, 74–82 10.1177/1754073910380973 21584224 PMC3093667

[B26] Dolan R. J. Vuilleumier P. (2003). Amygdala automaticity in emotional processing. Annal. N.Y. Acad. Sci. 985, 348–355 10.1111/j.1749-6632.2003.tb07093.x 12724170

[B27] Dolcos F. Diaz-Granados P. Wang L. McCarthy G. (2008). Opposing influences of emotional and non-emotional distracters upon sustained prefrontal cortex activity during a delayed-response working memory task. Neuropsychologia 46, 326–335 10.1016/j.neuropsychologia.2007.07.010 17765933

[B28] Dolcos F. McCarthy G. (2006). Brain systems mediating cognitive interference by emotional distraction. J. Neurosci. 26, 2072–2079 10.1523/JNEUROSCI.5042-05.2006 16481440 PMC6674921

[B29] Dux P. E. Ivanoff J. Asplund C. L. Marois R. (2006). Isolation of a central bottleneck of information processing with time-resolved fMRI. Neuron 52, 1109–1120 10.1016/j.neuron.2006.11.009 17178412 PMC2527865

[B30] Dvorak-Bertsch J. D. Curtin J. J. Rubinstein T. J. Newman J. P. (2007). Anxiety Moderates the Interplay Between Cognitive and Affective Processing. Psychol. Sci. 18, 699–705 10.1111/j.1467-9280.2007.01963.x 17680941 PMC3125602

[B31] Egner T. Etkin A. Gale S. Hirsch J. (2008). Dissociable neural systems resolve conflict from emotional versus nonemotional distracters. Cereb. Cortex 18, 1475–1484 10.1093/cercor/bhm179 17940084

[B32] Erthal F. De Oliveira L. Mocaiber I. Pereira M. Machado-Pinheiro W. Volchan E. (2005). Load-dependent modulation of affective picture processing. Cogn. Affect. Behav. Neurosci. 5, 388–395 10.3758/CABN.5.4.388 16541809

[B33] Essen D. C. V. Drury H. A. (1997). Structural and functional analyses of human cerebral cortex using a surface-based atlas. J. Neurosci. 17, 7079–7102 9278543 10.1523/JNEUROSCI.17-18-07079.1997 PMC6573261

[B34] Etkin A. Egner T. Kalisch R. (2011). Emotional processing in anterior cingulate and medial prefrontal cortex. Trends Cogn. Sci. 15, 85–93 10.1016/j.tics.2010.11.004 21167765 PMC3035157

[B35] Etkin A. Prater K. E. Hoeft F. Menon V. Schatzberg A. F. (2010). Failure of anterior cingulate activation and connectivity with the amygdala during implicit regulation of emotional processing in generalized anxiety disorder. Am. J. Psychiatry 167, 545–554 10.1176/appi.ajp.2009.09070931 20123913 PMC4367202

[B36] Etkin A. Prater K. E. Schatzberg A. F. Menon V. Greicius M. D. (2009). Disrupted amygdalar subregion functional connectivity and evidence of a compensatory network in generalized anxiety disorder. Arch. Gen. Psychiatry 66, 1361–1372 10.1001/archgenpsychiatry.2009.104 19996041 PMC12553334

[B36a] Etkin A. Wager T. D. (2007). Functional Neuroimaging of Anxiety: A Meta-Analysis of Emotional Processing in PTSD, Social Anxiety Disorder, and Specific Phobia. Am. J. Psychiatry. 164, 1476–1488 10.1176/appi.ajp.2007.07030504 17898336 PMC3318959

[B37] Eysenck M. W. Derakshan N. Santos R. Calvo M. G. (2007). Anxiety and cognitive performance: attentional control theory. Emotion 7, 336–353 10.1037/1528-3542.7.2.336 17516812

[B38] Fales C. Barch D. Burgess G. Schaefer A. Mennin D. Gray J. (2008). Anxiety and cognitive efficiency: Differential modulation of transient and sustained neural activity during a working memory task. Cogn. Affect. Behav. Neurosci. 8, 239–253 10.3758/CABN.8.3.239 18814461

[B39] Foland L. C. Altshuler L. L. Bookheimer S. Y. Eisenberger N. Townsend J. Thompson P. M. (2008). Evidence for deficient modulation of amygdala response by prefrontal cortex in bipolar mania. Psychiatry Res. Neuroimaging 162, 27–37 10.1016/j.pscychresns.2007.04.007 18063349 PMC2410029

[B40] Freese J. L. Amaral D. G. (2009). Neuroanatomy of the primate amygdala in The Human Amygdala, eds Whalen P. J. Phelps E. A. (New York, NY: The Guilford Press), 3–42

[B41] Friston K. J. Harrison L. Penny W. (2003). Dynamic causal modelling. Neuroimage 19, 1273–1302 10.1016/S1053-8119(03)00202-7 12948688

[B42] Ghashghaei H. T. Barbas H. (2002). Pathways for emotion: interactions of prefrontal and anterior temporal pathways in the amygdala of the rhesus monkey. Neuroscience 115, 1261–1280 10.1016/S0306-4522(02)00446-3 12453496

[B43] Goldin P. R. McRae K. Ramel W. Gross J. J. (2008). The neural bases of emotion regulation: reappraisal and suppression of negative emotion. Biol. Psychiatry 63, 577–586 10.1016/j.biopsych.2007.05.031 17888411 PMC2483789

[B44] Herath P. Klingberg T. Young J. Amunts K. Roland P. (2001). Neural correlates of dual task interference can be dissociated from those of divided attention: an fMRI Study. Cereb. Cortex 11, 796–805 10.1093/cercor/11.9.796 11532885

[B23] Hugdahl K. (1995). Dichotic Listening: probing temporal lobe functional integrity, in Brain Asymmetry, eds Davidson R. J. Hugdahl K. (Cambridge, MA: MIT Press), 123–156

[B45] Hugdahl K. Westerhausen R. Alho K. Medvedev S. Laine M. Hamalainen H. (2009). Attention and cognitive control: unfolding the dichotic listening story. Scand. J. Psychol. 50, 11–22 10.1111/j.1467-9450.2008.00676.x 18705670

[B46] Ikkai A. Curtis C. E. (2011). Common neural mechanisms supporting spatial working memory, attention and motor intention. Neuropsychologia 49, 1428–1434 10.1016/j.neuropsychologia.2010.12.020 21182852 PMC3081523

[B47] Jenkinson M. Bannister P. Brady M. Smith S. (2002). Improved optimization for the robust and accurate linear registration and motion correction of brain images. Neuroimage 17, 825–841 10.1006/nimg.2002.1132 12377157

[B48] Jenkinson M. Smith S. (2001). A global optimisation method for robust affine registration of brain images. Med. Image Anal. 5, 143–156 10.1016/S1361-8415(01)00036-6 11516708

[B49] Johnstone T. Van Reekum C. M. Urry H. L. Kalin N. H. Davidson R. J. (2007). Failure to regulate: counterproductive recruitment of top-down prefrontal-subcortical circuitry in major depression. J. Neurosci. 27, 8877–8884 10.1523/JNEUROSCI.2063-07.2007 17699669 PMC6672169

[B50] Joormann J. Siemer M. Gotlib I. H. (2007). Mood regulation in depression: differential effects of distraction and recall of happy memories on sad mood. J. Abnorm. Psychol. 116, 484–490 10.1037/0021-843X.116.3.484 17696704

[B51] Kalin N. H. Shelton S. E. Davidson R. J. (2004). The role of the central nucleus of the amygdala in mediating fear and anxiety in the primate. J. Neurosci. 24, 5506–5515 10.1523/JNEUROSCI.0292-04.2004 15201323 PMC6729317

[B52] Kerns J. G. (2006). Anterior cingulate and prefrontal cortex activity in an FMRI study of trial-to-trial adjustments on the Simon task. Neuroimage 33, 399–405 10.1016/j.neuroimage.2006.06.012 16876434

[B53] Kerns J. G. Cohen J. D. MacDonald A. W. Cho R. Y. Stenger V. A. Carter C. S. (2004). Anterior cingulate conflict monitoring and adjustments in control. Science 303, 1023–1026 10.1126/science.1089910 14963333

[B54] Kiehl K. A. Liddle P. F. Hopfinger J. B. (2000). Error processing and the rostral anterior cingulate: an event-related fMRI study. Psychophysiology 37, 216–223 10.1111/1469-8986.3720216 10731771

[B55] Kim C. Kroger J. K. Kim J. (2011). A functional dissociation of conflict processing within anterior cingulate cortex. Hum. Brain Mapp. 32, 304–312 10.1002/hbm.21020 21229616 PMC6869912

[B56] King J. A. Korb F. M. von Cramon D. Y. Ullsperger M. (2010). Post-error behavioral adjustments are facilitated by activation and suppression of task-relevant and task-irrelevant information processing. J. Neurosci. 30, 12759–12769 10.1523/JNEUROSCI.3274-10.2010 20861380 PMC6633589

[B57] Krug M. K. Carter C. S. (2010). Adding fear to conflict: a general purpose cognitive control network is modulated by trait anxiety. Cogn. Affect. Behav. Neurosci. 10, 357–371 10.3758/CABN.10.3.357 20805537

[B58] Lavie N. Hirst A. De Fockert J. W. Viding E. (2004). Load theory of selective attention and cognitive control. J. Exp. Psychol. Gen. 133, 339–354 10.1037/0096-3445.133.3.339 15355143

[B59] LeDoux J. (2003). The emotional brain, fear, and the amygdala. Cell. Mol. Neurobiol. 23, 727–738 14514027 10.1023/A:1025048802629 PMC11530156

[B60] Lévesque J. Eugène F. Joanette Y. Paquette V. Mensour B. Beaudoin G. (2003). Neural circuitry underlying voluntary suppression of sadness. Biol. Psychiatry 53, 502–510 10.1016/S0006-3223(02)01817-6 12644355

[B60a] MacDonald A. W. Cohen J. D. Stenger V. A. Carter C. S. (2000). Dissociating the Role of the Dorsolateral Prefrontal and Anterior Cingulate Cortex in Cognitive Control. Science. 288, 1835–1838 10.1126/science.288.5472.1835 10846167

[B61] Manoach D. White N. Lindgren K. Heckers S. Coleman M. Dubal S. (2004). Hemispheric specialization of the lateral prefrontal cortex for strategic processing during spatial and shape working memory. Neuroimage 21, 894–903 10.1016/j.neuroimage.2003.10.025 15006656

[B61a] Mauss I. B. Bunge S. A. Gross J. J. (2007). Automatic Emotion Regulation. Soc. Pers. Psychol. Compass, 1, 146–167 10.1111/j.1751-9004.2007.00005.x

[B62] Menon V. Adleman N. E. White C. D. Glover G. H. Reiss A. L. (2001). Error-related brain activation during a Go/NoGo response inhibition task. Hum. Brain Mapp. 12, 131–143 10.1002/1097-0193(200103)12:3<131::AID-HBM1010>3.0.CO;2-C 11170305 PMC6872006

[B63] Mincic A. M. (2010). Neural substrate of the cognitive and emotional interference processing in healthy adolescents. Acta Neurobiol. Exp. 70, 406–422 21196949 10.55782/ane-2010-1813

[B66] Morey R. D. (2008). Confidence intervals from normalized data: a correction to Cousineau (2005). Reason 4, 61–64

[B67] Moritz S. Birkner C. Kloss M. Jahn H. Hand I. Haasen C. (2002). Executive functioning in obsessive–compulsive disorder, unipolar depression, and schizophrenia. Arch. Clin. Neuropsychol. 17, 477–483 10.1016/S0887-6177(01)00130-5 14592001

[B68] Morris J. S. DeGelder B. Weiskrantz L. Dolan R. J. (2001). Differential extrageniculostriate and amygdala responses to presentation of emotional faces in a cortically blind field. Brain 124, 1241–1252 10.1093/brain/124.6.1241 11353739

[B69] Morris J. S. Friston K. J. Büchel C. Frith C. D. Young A. W. Calder A. J. (1998). A neuromodulatory role for the human amygdala in processing emotional facial expressions. Brain 121, 47–57 10.1093/brain/121.1.47 9549487

[B70] Northoff G. Heinzel A. Bermpohl F. Niese R. Pfennig A. Pascual-Leone A. (2004). Reciprocal modulation and attenuation in the prefrontal cortex: an fMRI study on emotional–cognitive interaction. Hum. Brain Mapp. 21, 202–212 10.1002/hbm.20002 14755839 PMC6871994

[B71] Ochsner K. N. Bunge S. A. Gross J. J. Gabrieli J. D. E. (2002). Rethinking feelings: an fMRI study of the cognitive regulation of emotion. J. Cogn. Neurosci. 14, 1215–1229 10.1162/089892902760807212 12495527

[B72] Ochsner K. N. Gross J. J. (2008). Cognitive emotion regulation insights from social cognitive and affective neuroscience. Curr. Direct. Psychol. Sci. 17, 153–158 10.1111/j.1467-8721.2008.00566.x PMC4241349 25425765

[B73] Ochsner K. N. Hughes B. Robertson E. R. Cooper J. C. Gabrieli J. D. E. (2008). Neural systems supporting the control of affective and cognitive conflicts. J. Cogn. Neurosci. 21, 1841–1854 10.1162/jocn.2009.21129 18823233 PMC6558970

[B74] Oei N. Y. L. Veer I. M. Wolf O. T. Spinhoven P. Rombouts S. A. R. B. Elzinga B. M. (2012). Stress shifts brain activation towards ventral “affective” areas during emotional distraction. Soc. Cogn. Affect. Neurosci. 7, 403–412 10.1093/scan/nsr024 21498384 PMC3324570

[B75] Paelecke-Habermann Y. Pohl J. Leplow B. (2005). Attention and executive functions in remitted major depression patients. J. Affect. Disord. 89, 125–135 10.1016/j.jad.2005.09.006 16324752

[B76] Penny W. D. Stephan K. E. Daunizeau J. Rosa M. J. Friston K. J. Schofield T. M. (2010). Comparing Families of Dynamic Causal Models. PLoS Comput. Biol. 6:e1000709 10.1371/journal.pcbi.1000709 20300649 PMC2837394

[B77] Pessoa L. Kastner S. Ungerleider L. G. (2002). Attentional control of the processing of neutral and emotional stimuli. Cogn. Brain Res. 15, 31–45 10.1016/S0926-6410(02)00214-8 12433381

[B78] Pessoa L. Kastner S. Ungerleider L. G. (2003). Neuroimaging studies of attention: from modulation of sensory processing to top-down control. J. Neurosci. 23, 3990–3998 12764083 10.1523/JNEUROSCI.23-10-03990.2003 PMC6741071

[B79] Peterson B. S. Kane M. J. Alexander G. M. Lacadie C. Skudlarski P. Leung H. C. (2002). An event-related functional MRI study comparing interference effects in the Simon and Stroop tasks. Cogn. Brain Res. 13, 427–440 10.1016/S0926-6410(02)00054-X 11919006

[B80] Phillips M. L. Drevets W. C. Rauch S. L. Lane R. (2003). Neurobiology of emotion perception II: implications for major psychiatric disorders. Biol. Psychiatry 54, 515–528 10.1016/S0006-3223(03)00171-9 12946880

[B81] Porrino L. J. Crane A. M. Goldman-Rakic P. S. (1981). Direct and indirect pathways from the amygdala to the frontal lobe in rhesus monkeys. J. Comp. Neurol. 198, 121–136 10.1002/cne.901980111 6164704

[B82] Postle B. R. (2006). Distraction-spanning sustained activity during delayed recognition of locations. Neuroimage 30, 950–962 10.1016/j.neuroimage.2005.10.018 16413797

[B83] Ridderinkhof K. R. Ullsperger M. Crone E. A. Nieuwenhuis S. (2004a). The role of the medial frontal cortex in cognitive control. Science 306, 443–447 10.1126/science.1100301 15486290

[B84] Ridderinkhof K. R. van den Wildenberg W. P. M. Segalowitz S. J. Carter C. S. (2004b). Neurocognitive mechanisms of cognitive control: the role of prefrontal cortex in action selection, response inhibition, performance monitoring, and reward-based learning. Brain Cogn. 56, 129–140 10.1016/j.bandc.2004.09.016 15518930

[B85] Sack A. T. (2009). Parietal cortex and spatial cognition. Behav. Brain Res. 202, 153–161 10.1016/j.bbr.2009.03.012 19463696

[B86] Schaefer S. M. Jackson D. C. Davidson R. J. Aguirre G. K. Kimberg D. Y. Thompson-Schill S. L. (2002). Modulation of amygdalar activity by the conscious regulation of negative emotion. J. Cogn. Neurosci. 14, 913–921 10.1162/089892902760191135 12191458

[B87] Shackman A. J. Sarinopoulos I. Maxwell J. S. Pizzagalli D. A. Lavric A. Davidson R. J. (2006). Anxiety selectively disrupts visuospatial working memory. Emotion 6, 40 10.1037/1528-3542.6.1.40 16637749

[B88] Shackman, Alexander J. Salomons T. V. Slagter H. A. Fox A. S. Winter J. J. Davidson R. J. (2011). The integration of negative affect, pain and cognitive control in the cingulate cortex. Nat. Rev. Neurosci. 12, 154–167 10.1038/nrn2994 21331082 PMC3044650

[B89] Scherer K. R. (2000). Psychological models of emotion in The Neuropsychology of Emotion, ed Borod J. C. (Oxford; New York: Oxford University Press), 137–162

[B90] Shin L. M. Whalen P. J. Pitman R. K. Bush G. Macklin M. L. Lasko N. B. (2001). An fMRI study of anterior cingulate function in posttraumatic stress disorder. Biol. Psychiatry 50, 932–942 10.1016/S0006-3223(01)01215-X 11750889

[B91] Siegle G. J. Thompson W. Carter C. S. Steinhauer S. R. Thase M. E. (2007). Increased amygdala and decreased dorsolateral prefrontal BOLD responses in unipolar depression: related and independent features. Biol. Psychiatry 61, 198–209 10.1016/j.biopsych.2006.05.048 17027931

[B92] Smith S. M. (2002). Fast robust automated brain extraction. Hum. Brain Mapp. 17, 143–155 10.1002/hbm.10062 12391568 PMC6871816

[B93] Steinhauer S. R. Hakerem G. (1992). The pupillary response in cognitive psychophysiology and schizophreniaa. Ann. N.Y. Acad. Sci. 658, 182–204 10.1111/j.1749-6632.1992.tb22845.x 1497258

[B94] Stevens M. C. Kiehl K. A. Pearlson G. D. Calhoun V. D. (2007). Functional neural networks underlying response inhibition in adolescents and adults. Behav. Brain Res. 181, 12–22 10.1016/j.bbr.2007.03.023 17467816 PMC2266817

[B95] Stout D. M. Shackman A. J. Larson C. L. (2013). Failure to filter: anxious individuals show inefficient gating of threat from working memory. Front. Hum. Neurosci. 7:58 10.3389/fnhum.2013.00058 23459454 PMC3586709

[B96] Taylor S. F. Phan K. L. Decker L. R. Liberzon I. (2003). Subjective rating of emotionally salient stimuli modulates neural activity. Neuroimage 18, 650–659 10.1016/S1053-8119(02)00051-4 12667842

[B97] Taylor S. F. Liberzon I. (2007). Neural correlates of emotion regulation in psychopathology. Trends Cogn. Sci. 11, 413–418 10.1016/j.tics.2007.08.006 17928261

[B97a] Urry H. L. van Reekum C. M. Johnstone T. Davidson R. J. (2009). Individual differences in some (but not all) medial prefrontal regions reflect cognitive demand while regulating unpleasant emotion. Neuroimage, 47, 852–863 10.1016/j.neuroimage.2009.05.069 19486944 PMC2766667

[B98] Van Dillen L. F. Heslenfeld D. J. Koole S. L. (2009). Tuning down the emotional brain: An fMRI study of the effects of cognitive load on the processing of affective images. Neuroimage 45, 1212–1219 10.1016/j.neuroimage.2009.01.016 19349235

[B99] Van Dillen L. F. Koole S. L. (2009). How automatic is “automatic vigilance”? The role of working memory in attentional interference of negative information. Cogn. Emotion 23, 1106–1117 10.1080/02699930802338178

[B100] Vogt B. A. Finch D. M. Olson C. R. (1992). Functional heterogeneity in cingulate cortex: the anterior executive and posterior evaluative regions. Cereb. Cortex 2, 435–443 10.1093/cercor/2.6.435-a 1477524

[B101] Vuilleumier P. Armony J. L. Clarke K. Husain M. Driver J. Dolan R. J. (2002). Neural response to emotional faces with and without awareness: event-related fMRI in a parietal patient with visual extinction and spatial neglect. Neuropsychologia 40, 2156–2166 10.1016/S0028-3932(02)00045-3 12208011

[B102] Vytal K. Cornwell B. Arkin N. Grillon C. (2012). Describing the interplay between anxiety and cognition: from impaired performance under low cognitive load to reduced anxiety under high load. Psychophysiology 49, 842–852 10.1111/j.1469-8986.2012.01358.x 22332819 PMC3345059

[B103] Wager T. D. Smith E. E. (2003). Neuroimaging studies of working memory. Cogn. Affect. Behav. Neurosci. 3, 255–274 15040547 10.3758/cabn.3.4.255

[B104] Whalen P. J. Rauch S. L. Etcoff N. L. McInerney S. C. Lee M. B. Jenike M. A. (1998). Masked presentations of emotional facial expressions modulate amygdala activity without explicit knowledge. J. Neurosci. 18, 411–418 9412517 10.1523/JNEUROSCI.18-01-00411.1998 PMC6793390

[B105] Yarkoni T. Poldrack R. A. Nichols T. E. Essen D. C. V. Wager T. D. (2011). Large-scale automated synthesis of human functional neuroimaging data. Nat. Methods 8, 665–670 10.1038/nmeth.1635 21706013 PMC3146590

[B106] Worsley K. J. (2001). Statistical analysis of activation images, in Ch 14, Functional MRI: An Introduction to Methods, eds Jezzard P. Matthews P. M. Smith S. M. (Oxford: Oxford University Press), 251–270

